# Hyper-N-glycosylated SEL1L3 as auto-antigenic B-cell receptor target of primary vitreoretinal lymphomas

**DOI:** 10.1038/s41598-024-60169-5

**Published:** 2024-04-26

**Authors:** Michelle Elbert, Frank Neumann, Maximilian Kiefer, Konstantinos Christofyllakis, Benedikt Balensiefer, Igor Kos, Gabi Carbon, Dominic Kaddu-Mulindwa, Joerg Thomas Bittenbring, Natalie Fadle, Evi Regitz, Falko Fend, Irina Bonzheim, Lorenz Thurner, Moritz Bewarder

**Affiliations:** 1 Internal Medicine I, Saarland University Medical Center, Homburg, Germany; 2 Institute of Pathology and Neuropathology and Comprehensive Cancer Center Tuebingen, Eberhard-Karls-University, Tuebingen, Germany

**Keywords:** B-cell receptor antigens, Primary vitreoretinal lymphoma, SEL1L3, Primary CNS lymphoma, SAMD14/neurabin-I, Auto-antigens, CNS cancer, Eye cancer, Haematological cancer, Non-hodgkin lymphoma, Translational research

## Abstract

Primary vitreoretinal lymphoma (PVRL) is a rare subtype of DLBCL and can progress into primary central nervous system lymphoma (PCNSL). To investigate the role of chronic antigenic stimulation in PVRL, we cloned and expressed B-cell receptors (BCR) from PVRL patients and tested for binding against human auto-antigens. SEL1L3, a protein with multiple glycosylation sites, was identified as the BCR target in 3/20 PVRL cases. SEL1L3 induces proliferation and BCR pathway activation in aggressive lymphoma cell lines. Moreover, SEL1L3 conjugated to a toxin killed exclusively lymphoma cells with respective BCR-reactivity. Western Blot analysis indicates the occurrence of hyper-N-glycosylation of SEL1L3 at aa 527 in PVRL patients with SEL1L3-reactive BCRs. The BCR of a PVRL patient with serum antibodies against SEL1L3 was cloned from a vitreous body biopsy at diagnosis and of a systemic manifestation at relapse. VH4-04*07 was used in both lymphoma manifestations with highly conserved CDR3 regions. Both BCRs showed binding to SEL1L3, suggesting continued dependence of lymphoma cells on antigen stimulation. These results indicate an important role of antigenic stimulation by post-translationally modified auto-antigens in the genesis of PVRL. They also provide the basis for a new treatment approach targeting unique lymphoma BCRs with ultimate specificity.

## Introduction

Primary vitreoretinal lymphomas (PVRL) are aggressive extranodal non-Hodgkin lymphomas restricted to the vitreous body and retina without evidence of systemic involvement and an estimated incidence below 0.05 per 100,000 per year. In the upcoming 5th edition of the World Health Organization Classification of Haematolymphoid Tumours (WHO-HAEM5), PVRL are grouped with large B-cell lymphomas involving the central nervous system (CNS) or testes to make up the entity of Primary large B-cell lymphoma of immune-privileged sites^1^. PVRL is the most common form of intraocular lymphoma and secondary CNS involvement occurs in up to 90% during the course of the disease while systemic dissemination is rare^2^. The underlying reasons for the selective tropism that restrict PVRL to the eye and CNS are still unclear. It is thought that a distinct chemokine milieu in combination with expression of the corresponding chemokine receptors by the malignant cells (e.g. CXCL12/CXCL13 and their receptors CXCR4/CXCR5) both contribute to the survival of B-cells in and chemotaxis to the CNS/eye^3,4^. In addition, eye and brain are thought to be immune-privileged sites where regular immune surveillance mechanisms are inactive, potentially promoting the development of malignant cells^5^. Primary CNS lymphoma (PCNSL) and PVRL are closely related diseases and share many genetic alterations like mutations in myeloid-derived factor 88 (MYD88) and CD79B leading to NF-κB pathway activation and homozygous loss of CDKN2A resulting in genomic instability. PCNSL and PVRL both usually belong to the molecularly defined MCD/C5 subtypes of DLBCL and show high rates of somatic hypermutation (SHM) of the rearranged immunoglobulin heavy chain (IGH) genes^6^.

The overrepresentation of the immunoglobulin heavy chain variable V region 4-34 (IGHV4-34), the expression of a functional B-cell receptor (BCR) despite ongoing somatic hypermutation and activation of the nuclear factor Kappa B (NF-kB) pathway indicate chronic stimulation of the BCR pathway by antigens^7,8^. In addition, the cause of the exclusive tropism of PCNSLs and PVRLs with restriction to the CNS/eye, where no germinal centers can be found, has not been fully elucidated. The interaction of PCNSL/PVRL BCRs with CNS/eye antigens is suspected to contribute to this phenomenon in addition to the decreased immune-surveillance which is characteristic for the CNS. In a recent study, PCNSL BCRs were found to bind to proteins that are predominantly expressed in the CNS with galectin-3 being the most prominent example^9^. Similarly, the proteins SAMD-14 and neurabin-I have been identified as auto-antigenic targets of 8/12 recombinantly expressed BCRs from PCNSLs^10^. Both proteins are preferentially expressed in the CNS and hyper-N-glycosylation is thought to be responsible for their auto-immunogenicity leading to a chronic immune-reaction. SAMD14/neurabin-I autoantibodies were frequently found in PCNSL patients further underscoring the notion of an autoimmune-reaction against SAMD14/neurabin-I as trigger for some PCNSLs. Post-translationally modified antigens Ars2 (hypophosphorylated) and LRPAP1 (unknown modification) have also been implicated to play an important role in the pathogenesis of ABC-type diffuse large B-cell lymphomas (DLBCL) and mantle cell lymphoma, respectively^11,12^. Belhouachi et al.^13^ reported on a remarkably restricted immunoglobulin gene repertoire for PVRLs and used PCNSL cases as a control group. Using published data from this study, we aimed to clone and express PVRL and PCNSL BCRs from available immunoglobulin heavy chain variable region (IGHV) and immunoglobulin light chain variable region (IGLV) DNA sequences as Fabs to test for SAMD14/neurabin-I reactivity of PCNSL cases and to test for specific binding of PVRL Fabs against human antigens.

## Methods

### Ethics approval and consent to participate

The study had been approved by the local ethics committee (Ärztekammer des Saarlandes) under the identification number: Ha 124/21. It was conducted according to the Declaration of Helsinki. Blood samples and tissue biopsy material were collected following written informed consent. The informed consent was obtained from all participants for study participation.

### Cells and cell lines

HEK 293T (for eukaryotic production of antigens/epitopes) cells and the DLBCL cell line OCI-Ly3 were purchased from DSMZ (Braunschweig, Germany). Cells of the DLBCL cell line TMD8 were obtained from the Department of Hematology of Göttingen Medical School (Göttingen, Germany). Lymphoblastoid cell lines (LCLs) were generated from two PVRL patients, one PVRL patient without SEL1L3 autoantibodies (PVRL LCL #1) and one PVRL patient with SEL1L3 autoantibodies (PVRL LCL #2) and healthy donors (HD LCL) by in-vitro infection of peripheral blood mononuclear cells (PBMCs) with Epstein-Barr-Virus (EBV). PBMCs were obtained by Ficoll density centrifugation (1500 rpm for 30 min.). Cells were cultured in RPMI 1640 medium (Pan Biotech, Aidenbach, Germany) and supplemented with 4 mmol glutamine and 10% FCS.

### Expression of SEL1L3-reactive BCRs in OCI-Ly3 and TMD8 cells

In order to mimic PVRL in-vitro, DLBCL cell lines OCI-Ly3 and TMD8 were transfected with SEL1L3-reactive BCRs. TMD8 cells regularly express BCRs of unknown reactivity but for Oci-Ly3 cells, Ars2 has been identified as cognate auto-antigen^11^. A modified pRTS-1 vector comprising a heavy chain variable region (VH), heavy chain constant regions CH1-CH4, the transmembrane regions TM1 and TM2, a cytoplasmatic tail, a furin + 2A sequence, a light chain variable region (VL) and the light chain constant region was used for cloning of the recombinant BCRs^14,15^. VH and VL gene sequences were previously published by Belhouachi et al.^13^ and ordered from GeneCust^®^ (GeneCust, 5690 Ellange, Luxembourg). Three washing steps of OCI-Ly3 and TMD8 cells at a density of 2 × 10^7^/ml in FCS-free RPMI-1640 medium were performed before transfection. 2 × 10^6^ cells were transfected with 5 μg plasmid DNA using a Gen Pulser (Biorad) and a 2 mm cuvette (140V, 30 ms. pulses). After resting on ice for 3 min, the cells were cultured in RPMI-1640 medium with 20% FCS. Hygromycin at 250 μg/ml was used for selection. VH gene PCRs were used to confirm transfection.

### Cloning and expression of PVRL and PCNSL BCRs as antibody fragments (Fabs)

DNA sequences were obtained from recently published data by Belhouachi et al.^13^ For 20 of 48 PCNSL and 20 of 55 PVRL cases complete or nearly complete BCR sequences of both Ig heavy and light chain variable region genes were available. Germline sequences were used to re-extend missing segments at the 5′ and 3′ ends. DNA clones comprising a ApaLI restriction site, the complete light chain V gene, a κ- or λ-constant region gene, a ribosome binding site, a signal sequence, the heavy chain V gene and a BstEII restriction site were ordered from GeneCust® (GeneCust, 5690 Ellange, Luxembourg) in a pUC57 vector. DNA clones were inserted into a modified pCES-1 vector in front of a γ-1 constant region gene and Histidine-tag for Fab expression. Fabs were produced in TG1 strain *E. coli* and purified via the Histidine-tag using Talon beads (Takara Bio USA, Inc., Mountain View, CA, USA) as reported previously^16,17^.

### Screening for antigenic BCR targets

PVRL BCRs in Fab format were pooled to a concentration of 10 µg/mL each. This Fab pool was used to screen for antigenic binders on protein macroarrays (human cDNA expression library, engine GmbH Neuendorfstr. 17, 16,761 Hennigsdorf, Germany), as described previously^18–20^.

### Expression of the antigens SEL1L3, SAMD14/neurabin-I, LRPAP1, Ars2, SLP2, PC9, RpoC and galectin-3

BCR-binding epitopes of SEL1L3, SAMD14/neurabin-I, Ars2, SLP2, PC9 and LRPAP1 as well as the whole antigens RpoC and galectin-3 were cloned into a pSFI vector incorporating a C-terminal FLAG tag. SAMD14/neurabin-I, Ars2, SLP2, LRPAP1, RpoC and galectin-3 have previously been identified as BCR antigens of B-cell neoplasia^9,12,18,19,21,22^. DNA sequences of the SEL1L3, SAMD14/neurabin-I, Ars2, SLP2, PC9 and LRPAP1 epitopes were obtained from clones of the UniPEx human cDNA expression library (Bioscience, Dublin, Ireland)^18^. The galectin-3 gene was cloned from DNA of PCNSL tissue sections. The gene of the *moraxella catarrhalis* bacterial antigen RpoC was amplified from bacterial DNA. All antigens were recombinantly expressed in pSFI-transfected HEK293 cells under the control of a CMV promoter.

### Identification of the BCR-binding epitope of SEL1L3

For the determination of the PVRL-binding epitope of SEL1L3, SEL1L3 fragments of different lengths were produced with C-terminal FLAG tags. The initial SEL1L3 fragment obtained from the UniPEx human cDNA membrane (Bioscience, Dublin, Ireland) clone spanned from amino acids (aa) 406–604. The produced fragments spanned from aa 406–480, aa 460–541, aa 519–604, aa 564–604, aa 537–578 and from aa 519–559. Flag-tagged fragments were coated on ELISA plates as described in the supplement and PVRL-Fabs and PVRL patient sera were tested for binding. ELISAs were repeated until no binding was observed. With every new SEL1L3 antigen fragment that was tested by ELISA for binding against PVRL Fabs, the previously tested antigen fragment was used as positive control.

### Western blots of SEL1L3

Lysates of LCLs of PVRL patients and healthy donors were used for Western blot analysis. Rabbit IgG Anti-SEL1L3 antibody (ab154052, ABCAM, Cambridge, UK) and murine anti-FLAG-antibody were used as primary antibodies (detailed description in the supplement).

### De-glycosylation of SEL1L3

Lysates of patient- and donor-derived LCLs were treated with enzymatic de-glycosylation (i.e. PNGase F, Endo-O-Glycosidase, α-2[3, 6, 8, 9]-neuraminidase, position-specific β-1–4-galactosidase, and β-N-acetylglucosaminidase; EDEGLY-Kit, Sigma).

### Expression of immunotoxins

BCR-binding epitopes of SEL1L3, RpoC and Ars2 were recombinantly expressed in *E. coli* BL21 as immunotoxins consisting of the BCR-binding antigens/epitopes conjugated to Pseudomonas exotoxin A as described previously^23^. For SEL1L3/ETA’ constructs SEL1L3 amino acids 560–580 were used, for Ars2/ETA’ constructs aa 342 to 375 were conjugated to pseudomonas exotoxin A and for RpoC/ETA’ constructs the RpoC amino acids 851–865 were incorporated^11,21^.

### Flow cytometry

SEL1L3 and Ars2 antigens/epitopes were tested for binding to BCR-transfected TMD8 and OCI-Ly3 cells as well as LCL cells by flow cytometry. For this, TMD8 and Oci-Ly3 cells were transferred to FACS tubes und incubated with 50 µl of Ars2-Flag or SEL1L3-Flag constructs at a concentration of 2 µg/ml for 30 min. on ice. After a washing step, mouse anti-Flag antibody (Monoclonal ANTI-Flag M2 antibody, SIGMA) at a dilution of 1:500 was added and incubated on ice for 30 min. Anti-mouse FITC antibody (Fluorescein (FITC)-conjugated AffiniPure F(ab')2 Fragment Goat Anti-Mouse IgG (H + L), ImmunoResearch) was used as secondary antibody at a dilution of 1:200 (incubation for 30 min, on ice). Cells are again incubated on ice for 30 min and washed in PBS, then resuspended in 500 µl of cold (phosphate buffered saline) PBS and measured on a BD FACSCanto™ flow cytometer.

For the analysis of apoptosis and/or necrosis the Annexin V-FITC Apoptosis Detection Kit (Sigma-Aldrich) was used.

### Immunoglobulin variable region gene PCR

DNA was isolated from LCL cells and from 2 PVRL specimens of a locally treated PVRL patient (1 extracted from fresh vitreous vitreous fluid at initial diagnosis and 1 obtained at relapse from a formalin-fixed, paraffin-embedded biopsy of the mamma). DNA isolated from PVRL specimens was obtained from the Department of Pathology and Neuropathology, Eberhard-Karls-University, Tuebingen, Germany. To isolate DNA from LCL cells, the QIAmp DNA Blood Mini Kit (QIAGEN GmbH, Hilden, Germany) was used according to the manufacturer's instructions. Multiplex PCRs for VH, Vκ and Vλ were performed according to the publication of van Dongen et al.^24^ Obtained IGHV sequences were analyzed and compared by IMGT-V-Quest.

### Statistical analysis

Statistical significance of proliferation and cytotoxicity assays was analyzed by multiple t test, using GraphPad Prism 7 (Boston, MA 02110).

## Results

### Confirmation of SAMD14/neurabin-I as BCR antigen of PCNSL

For 20 of 48 PCNSL patients reported by Belhouachi et al.^13^, DNA sequences of light and heavy chain variable genes were completely available (Table 1). Fabs, derived from these sequences, were tested for specific binding against the common lymphoma BCR antigens SAMD14/neurabin-I, LRPAP1, RpoC and galectin-3 by ELISA.

**Table 1:** Characterization of recombinantly expressed B-cell receptors from PCNSL cases.

PCNSL-ID	SAMD14/Neurabin-I-reactive	IGHV	Homology to germline	IGLV	Homology to germline
PCNSL01	–	VH3-21*06	93.06%	VL3-19	94.57%
PCNSL02	–	VH4-34*02	89.47%	VK1-33	100%
PCNSL03	** + **	VH4-34*01	83.51%	VK1-17	94.62%
PCNSL04	–	VH3-7*01	80.90%	VK1-16	100%
PCNSL06	–	VH3-33	88.89%	VK5-2	99.20%
PCNSL07	–	VH1-18*04	88.19%	VK2-28	99.60%
PCNSL08	–	VH3-33	96.88%	VK2-28	99.64%
PCNSL14	–	VH3-48	82.64%	VK2-30	99.30%
PCNSL15	–	VH4-34*02	79.30%	VK3-20	93.97%
PCNSL17	–	VH4-31*03	86.94%	VL2-8	92.01%
PCNSL19	–	VH4-59	88.04%	VK4-1	94.34%
PCNSL22	** + **	VH5-51*01	94.10%	VL2-23	95.49%
PCNSL28	–	VH4-34	85.61%	VK3-20	90.60%
PCNSL30	–	VH4-34	80.35%	VK1-39	85.98%
PCNSL31	–	VH4-34	76.84%	VK4-1	91.80%
PCNSL39	–	VH3-48	96.53%	VK1-5	98.20%
PCNSL41	–	VH3-23	95.83%	VK2-28	89.60%
PCNSL45	–	VH4-34*01	93.33%	VK3-15	97.35%
PCNSL46	–	VH4-59	92.63%	VK3-20	96.25%
PCNSL47	–	VH4-34*01	83.80%	VK1-39	92.47%

**Figure 1:**
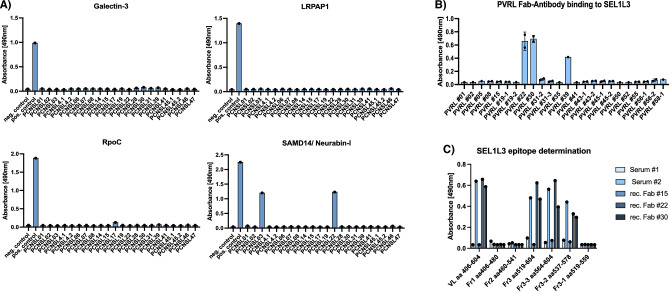
(**A)** ELISA to evaluate the affinity of 20 Fabs derived from published PCNSL cases (Belhouachi et al.)^13^ against the antigens SAMD14/Neurabin-I, LRPAP1, RpoC, and Galectin-3. Among tested Fabs, two showed reactivity to SAMD14/Neurabin-I. No discernible binding was observed between PCNSL Fabs and LRPAP1, RpoC, or Galectin-3. (**B**) Twenty PVRL-BCR sequences (Belhouachi et al.)^13^ were cloned and produced as Fab-antibodies in E. coli and screened for binding to human antigens on a UniPEx human cDNA expression library protein macroarray, identifying SEL1L3 as possible BCR target. ELISA assays showed binding of 3/20 PVRL Fabs (PVRLs #22, #30 and #39) to SEL1L3. (**C**) Reactivity of PVRL patient sera and recombinant Fabs #15, #22, #30 to SEL1L3 as it was expressed on the UniPEx membrane (aa 406–604, termed as full-length) and SEL1L3 fragments (Fr1: aa 406–480; Fr2: aa 460–541; Fr3: aa 519–604; Fr3-3: aa 564–604; Fr3-2: aa 537–578; Fr3-2: aa 519–559), assessed by ELISA. Patient serum #1 and recombinant Fab #15 show no reactivity to full-length SEL1L3 or its’ fragments. PVRL patient serum #2 and recombinant Fabs #22 and #30 are reactive to full-length SEL1L3. Binding properties to SEL1L3 fragments suggest an epitope region spanning approximately from aa 560 to aa 580. ELISAs of figure were repeated multiple times and figures (**A**–**C**) are exemplary. ELISAs of Fig. 2B were performed simultaneously in two wells, which is represented by two dots on top of the bars.

The PCNSL BCR-binding epitope of SAMD14 and neurabin-I was the specific target of 2/20 PCNSL-derived Fabs. In control experiments, none of the recombinantly expressed PCNSL-Fabs showed binding to LRPAP1, RpoC or galectin-3 (Fig. 1A).

### Protein SEL1L3 as target of BCRs derived from PVRL patients

PVRL Fab-antibodies were tested for affinity against the antigens SAMD14/Neurabin-I, LRPAP1, RpoC and Galectin-3 with no detectable binding. Subsequently, PVRL Fabs were used for screening on the UniPEx human cDNA membrane. By this, SEL1L3 was identified as a target of the tested PVRL BCRs. Specifically, SEL1L3 was identified as target of 3/20 PVRL Fabs, derived from PVRL-BCR sequences published by Belhouachi et al. (Fig. 1B)^13^. BCRs from PVRL specimens #22, #30 and #39 showed 4 – 7 × higher absorbance as non-reactive Fabs. All three SEL1L3-reactive PVRL BCRs share the use of the variable Ig gene segment IGHV4-34 (Table 2).

**Table 2:** Characterization of recombinantly expressed B-cell receptors from PVRL cases.

PVRL-ID	SEL1L3-reactive	IGHV	Homology to germline	IGLV	Homology to germline
PVRL 01	–	IGHV 4–34	90.5%	IGKV3-20	93.4%
PVRL 02	–	IGHV 4–34	81.8%	IGLV1-44	90.8%
PVRL 05	–	IGHV 4–34	86.3%	IGLV1-40	92.1%
PVRL 08	–	IGHV3-7*01	83%	IGKV1-39*01	98.4%
PVRL 15	–	IGHV4-34*02	83.2%	IGKV3-11*01	93.0%
PVRL 18	–	IGHV4-34*03	77.5%	IGKV3-20*01	87.6%
PVRL 19	–	IGHV 4–34	89.5%	IGKV3-20	92.7%
PVRL 22	+	IGHV4-34*01	85.6%	IGKV1-17*01	93.8%
PVRL 30	+	IGHV4-34*02	85.3%	IGKV3-11*01	94.1%
PVRL 31	–	IGHV3-7*01	86.1%	IGKV6-21*01	92.2%
PVRL 35	–	IGHV 4–34	83.5%	IGKV4-1	99.6%
PVRL 39	+	IGHV 4–34	85.3%	IGKV3-20	90.4%
PVRL 41	–	IGHV 4–34	87.7%	IGKV3-20	92.3%
PVRL 43	–	IGHV4-34*01	86.2%	IGKV3-20*01	91.5%
PVRL 45	–	IGHV4-34*01	86.0%	IGLV1-40*01	87.2%
PVRL 50	–	IGHV 4–34	87.0%	IGKV3-11	92.2%
PVRL 52	–	IGHV 4–34	81.1%	IGKV2-29	99.2%
PVRL 55	–	IGHV5-10*03	96.5%	IGLV3-1*01	91.8%
PVRL 56	–	IGHV4-34*02	82.8%	IGKV4-1*01	89.1%
PVRL 58 a	–	IGHV 4–34	86.3%	IGLV2-14	94.0%

To identify the PVRL BCR-binding epitope of SEL1L3, PVRL Fabs and patient sera were tested for binding to different fragments of SEL1L3 (Fig. 1C). The identified SEL1L3 clone of the UniPEx human cDNA membrane spans from amino acids 406–604. This fragment was further split into three overlapping fragments: fragment 1 (Fr1, aa 406–480), fragment 2 (Fr2, aa 460–541) and fragment 3 (Fr3, aa 519–604). SEL1L3-reactive BCRs #22 and #30 as well as SEL1L3-reactive patient serum bound specifically to Fr3. Fr3 was divided into the overlapping fragments Fr3-1 (aa 519–559), Fr3-2 (aa 537–578) and Fr3-3 (aa 564–604). PVRL-derived Fabs #22, #30 and patient serum #2 (serum from a PVRL patient with an SEL1L3-reactive lymphoma BCR) demonstrated binding to Fr3-3. Binding was weaker after incubation with Fr3-2 and abrogated for Fr3-1 (Fig. 1C).

### BCR-mediated targeting of lymphoma cells by SEL1L3 and SEL1L3-drug conjugate

**Figure 2:**
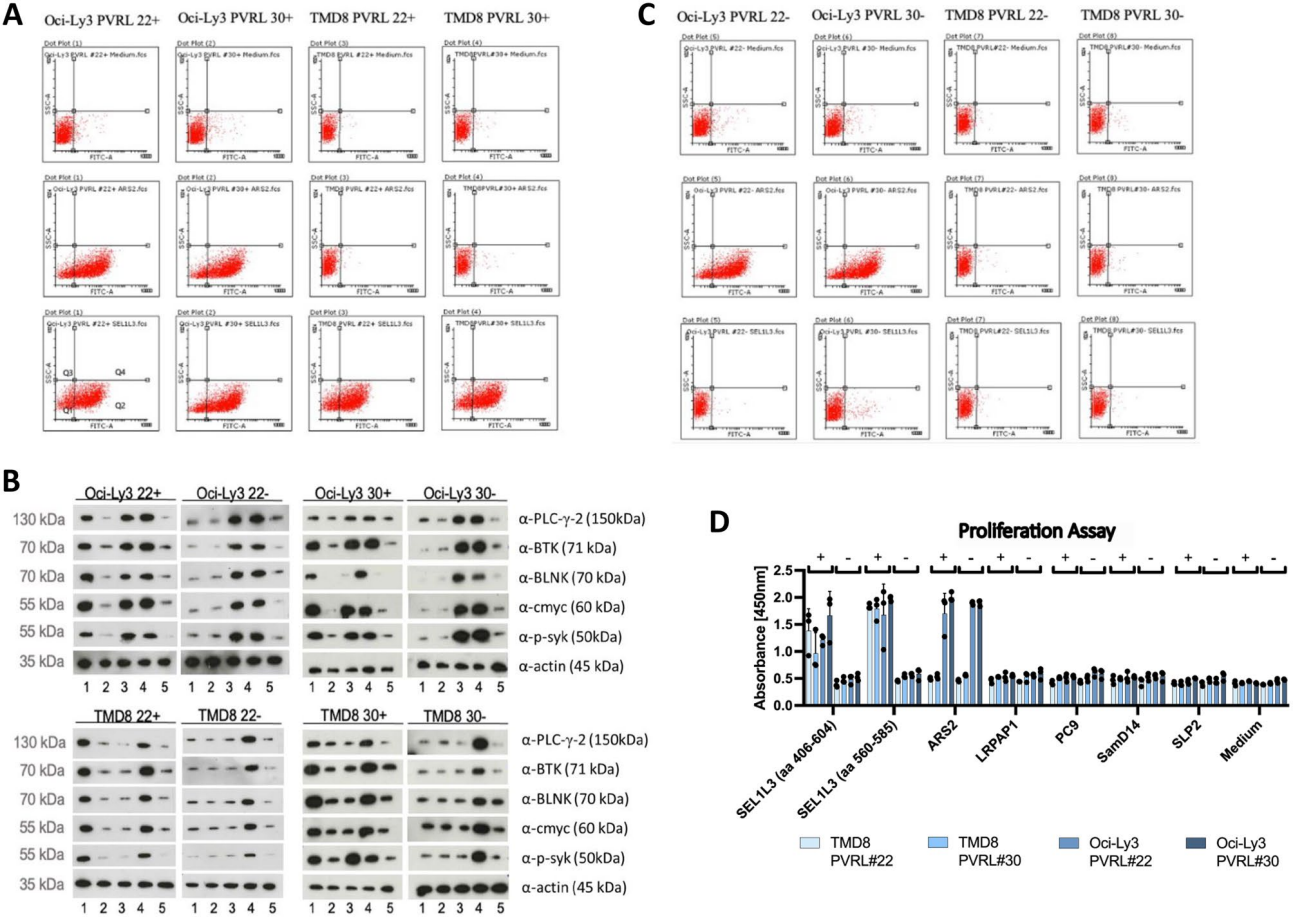
(**A**) DLBCL cell lines TMD8 and Oci-Ly3 were transfected with BCRs of PVRL cases #22 and #30. Both BCRs were previously identified as reactive to SEL1L3. TMD8 cells express BCRs of unknown reactivity as natural BCR. For Oci-Ly3 cells, Ars2 has been identified as cognate auto-antigen of their BCR. Expression of BCRs from PVRL cases #22 and #30 was induced with doxycycline ( +). Oci-Ly3 and TMD8 cells were incubated for 45 min with medium, Ars2-Flag or SEL1L3-Flag. After washing steps, FITC-A coupled anti-Flag antibody was used as secondary system. Ars2 shows binding to OCI-Ly3 cells but not to TMD8 cells regardless of the expression of SEL1L3-reactive BCRs (**A** and **B**). SEL1L3 shows binding to TMD8 and OCI-Ly3 cells after the expression of SEL1L3-reactive BCRs of PVRL cases #22 and #30 is switched on using doxycycline. (**B**) Without induction of BCR expression, no binding of SEL1L3 to either cell line is observed. (**C**) 12% SDS-Page Western blots of DLBCL cell line lysates. TMD8 and Oci-Ly3 cells transfected with an inducible SEL1L3-reactive BCR were incubated over night with SEL1L3, LRPAP1, Ars2, anti-IgM or medium. Expression of SEL1L3-reactive BCRs derived from PVRL patients #22 and #30 was activated the day before with doxycycline (induction of BCR expression is marked with +). In OCI-Ly3 cells, BCR pathway proteins are upregulated after incubation with their cognate BCR antigen Ars2 (3) and the positive control anti-IgM (4). For SEL1L3 (1) this effect was observed only after induction of a SEL1L3-reactive BCR. Medium (5) only or LRPAP1 (2) had no influence on the activation status of the BCR pathway of OCI-Ly3 cells. Similar results were observed for TMD8 cells with the exception that Ars2 had no effect on the upregulation of BCR signaling pathway proteins since TMD8 cells do not express an Ars2-reactive BCR. (**D**) Incubation with SEL1L3 epitope induces proliferation of DLBCL cell lines only after expression of patient-derived, SEL1L3-reactive BCRs (induction of BCR expression is marked with +) on TMD8 and Oci-Ly3 cells. Ars2 as cognate antigen of Oci-Ly3 BCRs was used as positive control. Proliferation experiments shown in 2D were performed in triplicate, i.e. simultaneously in three separate wells.

To test SEL1L3 as targeting moiety of constructs aiming at the BCR of PVRL cells, DLBCL cell lines TMD8 and Oci-Ly3 were transfected with BCRs of the PVRL cases #22 and #30, which were identified as reactive to SEL1L3 (Fig. 2).

Flow cytometry shows strong binding of SEL1L3 to OCI-Ly3 and TMD8 cells after expression of BCRs with SEL1L3-reactivity is induced (Fig. 2A). When the expression of respective BCRs is not induced, no binding of SEL1L3 to either cell lines can be detected (Fig. 2B). Quantitative flow cytometry measurements are summarized in Suppl. Table 1. Ars2 shows binding to OCI-Ly3 cells but not to TMD8 cells. Expression of SEL1L3-reactive BCRs had no influence on binding properties of OCI-Ly3 and TMD8 cells to Ars2 or medium.

Western Blot analyses of TMD8 and Oci-Ly3 cell lines expressing SEL1L3 reactive BCRs indicate strong activation of the BCR pathway after incubation with SEL1L3, demonstrated by upregulation of PLC-gamma-2, BTK, BLNK, cmyc and p-syk (Fig. 2C).

Proliferation-assays show that SEL1L3 protein induces proliferation of DLBCL cells only after expression of patient-derived SEL1L3-reactive BCRs (Fig. 2D). Ars2 as cognate antigen of Oci-Ly3 BCRs induced proliferation of OCI-Ly3 cells exclusively, while addition of LRPAP1, PC9, SamD14, SLP2 and medium had no effect on proliferation of either cell line.

**Figure 3:**
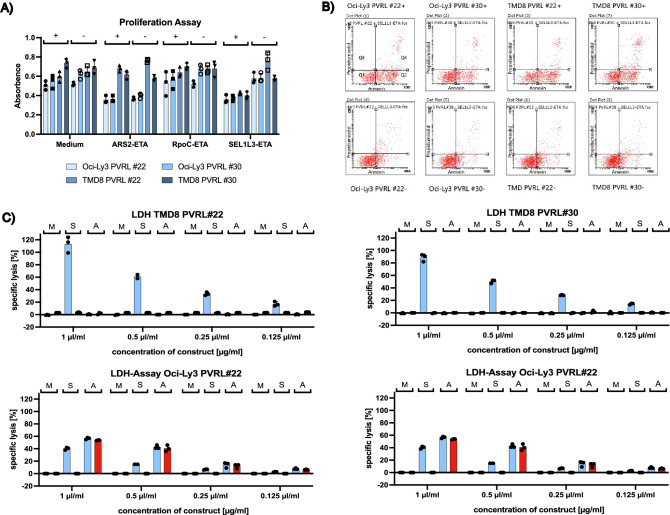
(**A**) DLBCL cell lines TMD8 and Oci-Ly3 were transfected with BCRs of PVRL cases #22 and #30. Both PVRL cases were previously identified as reactive to SEL1L3. TMD8 cells express BCRs of unknown reactivity as their natural BCR. For Oci-Ly3 cells, Ars2 has been identified as cognate auto-antigen of their BCR. The BCR binding epitope of SEL1L3 (aa 560–585) conjugated to a truncated form of Pseudomonas exotoxin A (ETA) reduces proliferation of lymphoma cells expressing the respective BCR. Ars2-ETA was used as positive control on Ars2-reactive OCI-Ly3 cells. Rpoc, an irrelevant antigen, conjugated to ETA’ was used as negative control. Expression of BCR was induced with doxycycline ( +). (**B**) Flow cytometric annexin assays using the Annexin V-FITC Apoptosis Detection Kit (Sigma-Aldrich) were performed to elucidate the mechanisms of cell death after treatment with SEL1L3 immunotoxins. OCI-LY3 and TMD8 cells transfected to express a BCR with reactivity against SEL1L3 were treated with Ars2-ETA, RpoC-ETA, SEL1L3-ETA (all at 0.5 µg/mL), staurosporine (1 µg/mL), or medium. After incubation with SEL1L3 immunotoxins both OCI-Ly3 and TMD8 cells undergo mainly apoptosis if expression of SEL1L3-reactive BCRs is switched on by doxycycline (upper row) and has no effect on both cell lines without prior induction of respective BCRs (bottom row). Quantitative measurements are shown in Suppl. Table 2. (**C**) LDH cytotoxicity assays of SEL1L3-ETA immunotoxins on Oci-Ly3 as well as TMD8 cells expressing SEL1L3-reactive BCRs derived from PVRL patients #22 and #30. Ars2-ETA constructs served as positive control as Ars2 is the cognate antigen of the constitutively expressed BCR in Oci-Ly3 cells. TMD8 cells express BCRs of unknown reactivity as their natural BCR. Bars are depicted in pairs, left bar in light blue (expression of SEL1L3-reactive BCRs turned on) and right bar in red (no expression of SEL1L3-reactive BCRs). Cells were incubated with medium, SEL1L3 or Ars2. Starting at doses of 0.125 µg/ml, SEL1L3-ETA constructs mediated dose-dependent and specific lysis of lymphoma cells (TMD8 and Oci-Ly3) only after doxycycline-induced expression of SEL1L3-reactive BCRs. Ars2-ETA constructs mediate cytotoxicity to Oci-Ly3 cells regardless of PVRL BCR expression and had no effect on TMD8 cells. Proliferation assays in Fig. 3A and LDH assays in Fig. 3C were performed in triplicate, i.e. simultaneously in three separate wells. *M* medium, *S* SEL1L3-ETA, *A* Ars2-ETA.

In addition, the therapeutic potential and killing properties of SEL1L3/Pseudomonas exotoxin immunotoxins (SEL1L3-ETA) were investigated. After incubation with SEL1L3-ETA, proliferation of OCI-Ly3 and TMD8 cells expressing a SEL1L3-reactive BCR was reduced (Fig. 3A). Ars2-ETA had similar effects on OCI-Ly3 cells regardless of expression of PVRL BCRs and had no effect on TMD8 cells. LDH cytotoxicity assays were performed to demonstrate the therapeutic potential of SEL1L3-ETA constructs (Fig. 3C). Starting from doses of 0.125 µg/ml, SEL1L3-ETA constructs mediate dose-dependent and specific lysis to lymphoma cells (TMD8 and Oci-Ly3) only after induction of expression of SEL1L3-reactive BCRs. Ars2-ETA constructs mediate cytotoxicity to Oci-Ly3 cells regardless of PVRL BCR expression and had no effect on TMD8 cells. To elucidate the mechanisms of cell death after treatment with SEL1L3 immunotoxins, flowcytometric annexin assays were performed (Fig. 3B). After incubation with SEL1L3 immunotoxins both OCI-Ly3 and TMD8 cells undergo mainly apoptosis if expression of SEL1L3-reactive BCRs is switched on by doxycycline. Quantitative measurements are shown in Suppl. Table 2.

### PVRL patient with SEL1L3-reactive lymphoma BCR

At our institution, we were able to identify one patient with PVRL expressing a SEL1L3-reactive BCR. The PVRL BCR of this patient was cloned and expressed as Fab from the initial vitreous body biopsy and from a specimen obtained at the time of relapse in the mamma. Serum of this patient showed high antibody titers against SEL1L3. Furthermore, LCL cells were generated from PBMCs of this patient (PVRL LCL #2).

**Figure 4:**
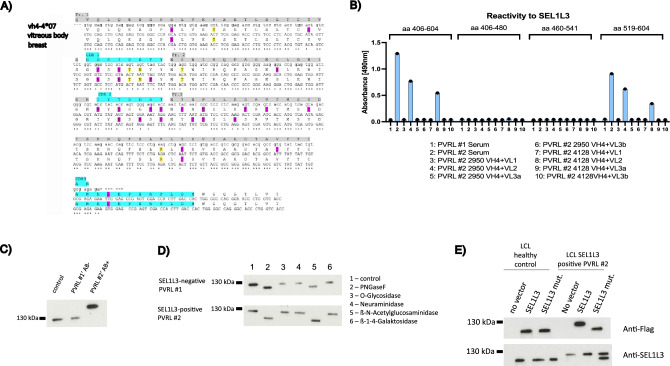
(**A**) DNA sequences of a PVRL case at first diagnosis and at relapse (obtained from a breast punch biopsy). VH4-04*07 was used in both lymphoma manifestations. Amino acids marked in pink and yellow (silent mutations) differ from the germline sequence. CDR regions are marked in light blue. Ig DNA sequences of the primary tumor and at relapse are largely consistent and show a striking conservation of the CDR3 region, indicating a continued dependence of lymphoma cells on BCR stimulation. (**B**) Lymphoma BCRs were cloned and expressed from both biopsies (BCR cloned from the eye at initial diagnosis: PVRL #2 2950, BCR cloned from breast biopsy at relapse: PVRL #2 4128) and showed binding to SEL1L3 in ELISA. Both BCRs use the same heavy and light chain (VH4 + VL2). Serum of respective patient (PVRL #2 Serum) was reactive to SEL1L3, serum of a control PVRL patient showed no binding to SEL1L3 (PVRL #1 Serum). (**C**) 10% SDS-PAGE Western blot analysis was performed on LCL lysates generated from a healthy control, an SEL1L3 antibody-negative PVRL patient, and an antibody-positive PVRL patient. SEL1L3 protein from the SEL1L3-positive PVRL patient exhibited a higher molecular mass (> 130 kDa) in comparison to SEL1L3 in the antibody-negative PVRL patient and the healthy control (< 130 kDa). (**D**) Upon treatment with N-acetyl-β-D-glucosaminidase and PNGase F, the difference in molecular weight vanishes, implicating post translational hyper-N-glycosylation of SEL1L3 as trigger of an auto-immune reaction in some PVRL patients. (**E**) 8% SDS-PAGE Western blot of LCL cells derived from a healthy donor and a PVRL patient with serum anti-SEL1L3 antibodies using Anti-Flag antibody and Anti-SEL1L3 antibodies. LCL cells were transfected with either wild type SEL1L3 or SEL1L3 mutated at amino acid 527, the suspected site of hyper-N-glycosylation. Western blots developed using the Anti-Flag antibody exclusively detect the transfected SEL1L3 protein, whereas Western blots developed with commercially available anti-SEL1L3 antibodies can detect both transfected and naturally expressed SEL1L3. In LCLs derived from a PVRL patient positive for SEL1L3 antibodies, the constitutively expressed SEL1L3 exhibits a higher molecular weight compared to the mutated SEL1L3 and SEL1L3 expressed in LCLs derived from a healthy control. Mutation of amino acid 527 leads to a lower molecular weight of transfected SEL1L3 in PVRL patient-derived LCLs compared to SEL1L3 expressed in the SEL1L3 auto-antibody positive PVRL patient. These findings suggest that the observed molecular weight difference in SEL1L3 between healthy individuals and certain PVRL patients can be attributed to hyper-N-glycosylation at aa 527.

IGHV sequence analysis from the PVRL at initial diagnosis and at relapse revealed clonal relation (VH4-4*07) and additionally showed a highly conserved CDR3 region at relapse (Fig. 4A). When cloned and expressed as Fabs, BCRs from both biopsies showed strong binding capacity to SEL1L3 in ELISA (Fig. 4B), indicating ongoing dependence on BCR pathway stimulation by cognate antigen, i.e. SEL1L3, of PVRL cells at relapse.

In Western Blot analysis of LCL cell lysates, SEL1L3 showed a prominent difference in molecular mass in patient PVRL #2’ (SEL1L3-reactive PVRL BCR) as compared to patient PVRL #1’ (not SEL1L3-reactive PVRL BCR) and healthy controls (Fig. 4C). In order to test for hyper-glycosylation as cause for the difference in molecular mass, a de-glycosylation kit was used (Fig. 4D). After N-glycosidase treatment of SEL1L3 obtained from LCL lysates from a PVRL patient with detectable SEL1L3 antibody titer, the molecular weight of SEL1L3 normalizes, indicating hyper-N-glycosylation of SEL1L3 as cause for its increased molecular weight.

Using the UniProt database, several possible glycosylation sites were identified with asparagine at position 527 being the closest one to the identified epitope^25^. To confirm aa 527 of SEL1L3 as the relevant site of hyper-N-glycosylation in PVRL patients, we used Western Blot analysis of LCL cells of a healthy control and of a SEL1L3-positive PVRL patient (PVRL #2’). LCLs were transfected with wild-type SEL1L3 and at aa 527 mutated SEL1L3. Anti-Flag developed Western Blots detect only transfected SEL1L3 (either wild-type or mutated). Western Blots developed with anti-SEL1L3 antibodies detect transfected as well as constitutionally expressed SEL1L3 (Fig. 4E). In LCLs derived from a PVRL patient with a SEL1L3-reactive lymphoma BCR, constitutionally expressed SEL1L3 shows a higher molecular mass than mutated SEL1L3 and SEL1L3 expressed in LCLs derived from a healthy control. Mutation of aa 527 leads to a lower molecular mass of transfected SEL1L3 in patient-derived LCLs compared to wild type SEL1L3. These results indicate that the difference in molecular weight of SEL1L3 between healthy and PVRL LCLs stems from hyper-N-glycosylation of aa 527 (asparagine) and that the cellular mechanisms leading to hyper-glycosylation of SEL1L3 in PVRL patients are ongoing in patient-derived LCLs.

**Figure 5:**
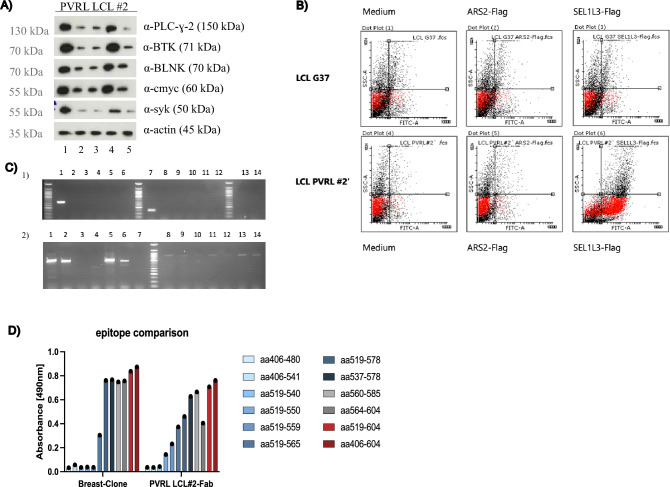
(**A**) PVRL LCL #2 cells (LCLs generated from a PVRL patient with SEL1L3-reactive lymphoma BCR and anti-SEL1L3 serum titer) were incubated over night with SEL1L3 (1), LRPAP1 (2), Ars2 (3), anti-IgM (4) or medium (5). Western blot analysis shows strong activation and upregulation of BCR pathway proteins after incubation with SEL1L3 and anti-IgM (positive control). LRPAP1, Ars2 and medium had no effect on BCR pathway activation. (**B**) In line with these results, more than 50% of PVRL LCL #2 cells also showed strong binding to SEL1L3 as assessed by flow cytometry. PVRL LCL #2 cells were incubated for 45 min with medium, Ars2-Flag or SEL1L3-Flag. After washing steps, FITC-A coupled anti-Flag antibody was used as secondary system. Quantitative measurements are shown in Suppl. Table 3. (**C**) Results of the immunoglobulin variable region gene PCR of PVRL patient-derived LCL cells with a SEL1L3-reactive BCR (PVRL LCL #2). VH1-2*02 and VK1-39*01 were identified as monoclonal heavy and light chain, respectively. Primers used in (1) and (2) in lanes 1–14 are shown in Suppl. Table 4. This is in contrast to the heavy chain variable region VH4-04*07, that was found in both lymphoma manifestations of this patient. (**D**) Epitope mapping for BCRs generated from PVRL LCL #2 and from biopsy at relapse was performed. LCLs were generated of the PVRL patient from whom the biopsy at relapse was taken. This patients lymphoma cells express BCRs with SEL1L3 reactivity. SEL1L3 epitopes differed between both BCRs indicating their different clonal origin.

In Western blot analyses PVRL LCL #2 cells show strong activation and upregulation of BCR pathway proteins after incubation with SEL1L3 (Fig. 5A). In line with these results, more than 50% of PVRL LCL #2 cells also showed strong binding to SEL1L3 as assessed by flow cytometry (Fig. 5B). Quantitative flow cytometry measurements are summarized in Suppl. Table 3. Immunoglobulin variable region gene PCR confirmed the presence of monoclonal B cells within PVRL LCL #2 cells (Fig. 5C) using VH1-2*02 and VK1-39*01 as Ig gene segments for BCR generation. The B-cell clone that emerged from generated patient LCLs has therefore no clonal relation to PVRL cells of this patient and seems to have emerged as second auto-reactive clone *in-vitro* out of immortalized polyclonal B-cells. Fabs generated from patient PVRL biopsies and from PVRL LCL #2 cells were used for comparison of epitopes. The SEL1L3 epitope recognized by the BCR generated from monoclonal PVRL LCL #2 cells differed from the epitope bound by the BCRs obtained from PVRL biopsies (Fig. 5D).

Flowcytometric annexin assays were able to show apoptosis of monoclonal PVRL LCL #2 cells after incubation with SEL1L3-ETA immunotoxin demonstrating the therapeutic potential of SEL1L3 incorporating constructs to treat lymphoma cells expressing SEL1L3-reactive BCRs (data not shown).

## Discussion

Restriction of the usage of immunoglobulin heavy chain variable region genes in B-cell lymphomas to one or few genes out of more than 100 IGHV genes indicates stimulation of the malignant clone by a shared common antigen. It is thought that identical or similar DNA sequences of the BCR result in structurally identical BCRs with closely related binding properties to shared common antigens. A chronic and misdirected immune reaction against a shared common immunogenic antigen may explain the IGHV restriction of some B-cell lymphomas. However, until now only few antigens of self or microbial origin have been identified for B-cell lymphomas^10,12,18,19,21,22,26–39^. Even more, none have been described as part of lymphomagenesis in PVRL despite a strikingly biased IGHV repertoire with usage of the IGHV4-34 gene in 63.6% of cases^6,13,40^. Screening for antigenic binders with PVRL BCRs is hampered by low incidence of PVRL and scarcity of specimens. Recently, Belhouachi et al.^13^ reported on a PVRL case series of 55 cases and published the corresponding VH and VL BCR sequences. We were able to clone and express 20/55 PVRL BCRs from this series as Fab antibodies (Table 2) and used these antibodies to screen for antigenic BCR targets on a protein macroarray.

To test our experimental approach and to verify previous results we additionally cloned and expressed 20 Fab-format BCRs from PCNSL cases that were also reported in the study of Belhouachi et al. For 20 of 48 PCNSL patients, VH and CL DNA sequences were available (Table 1). Fabs were then tested for specific binding against the common lymphoma BCR antigens SAMD14/neurabin-I, LRPAP1, RpoC and galectin-3 using ELISA^9,10,12,21,41^. The shared epitope of SAMD14 and neurabin-I has previously been described as auto-antigenic target of 8/12 (67%) recombinantly expressed PCNSL BCRs. In the current study, the PCNSL BCR-binding epitope of SAMD14 and neurabin-I was the specific target of 2/20 PCNSL-derived Fabs. In control experiments, none of the recombinantly expressed PCNSL-Fabs showed binding to LRPAP1, RpoC or galectin-3 (Fig. 1A). These results confirm SAMD14/neurabin-I as common auto-antigen of PCNSL BCRs but with a lower frequency as previously reported. Together with the initially reported PCNSLs, 32 recombinant PCNSL BCRs have been tested so far with 10/32 (31%) showing specific binding capacity towards SAMD14/neurabin-I.

Using PVRL Fabs we identified Protein sel-1 homolog 3 (SEL1L3) as the auto-antigenic target of 3/20 (15%) PVRL BCRs. After fragmentation of SEL1L3 and subsequent ELISAs, the BCR-reactive epitope was determined to span from aa 560–580. SEL1L3 is a 1132 aa containing protein encoded by the SEL1L3 gene on chromosome 4, which is expressed in many tissues, among others in rod photoreceptor cells of the eye and lymphoid tissue (https://www.proteinatlas.org)^25,42^. Since SEL1L3 is expressed ubiquitously, we believe that it plays no relevant role in the selective tropism of PVRL to the eye/CNS. The function of SEL1L3 is still not known but a recent study found that SEL1L3 might play a role in the regulation of cell stress^43^. Also, different roles of SEL1L3 in aging and the immune system are discussed^44,45^. Two out of three identified SEL1L3-reactive BCRs were transfected into DLBCL cell lines (OCI-Ly3 and TMD8) to express respective BCRs in addition to their biological BCRs. Using this model of PVRL cells, a functional role of the transfected BCRs with an increased growth rate after the addition of the cognate antigen SEL1L3 and the therapeutic potential of SEL1L3 incorporating immunotoxins could be demonstrated. Cell death of lymphoma cells expressing PVRL BCRs that were treated with SEL1L3 immunotoxins was mainly due to apoptosis and to a smaller degree due to necrosis. De-glycosylation experiments determined aa 527 (asparagine) as site of hyper-N-glycosylation, which we believe may have triggered a chronic immune reaction ultimately developing into PVRL. Similar post-translational modifications have been reported previously and were also suspected to play a role in the development of B-cell neoplasia^18,10,22,33,34,46^. We also report on two patients with PVRL, one expressing a BCR with reactivity against SEL1L3 and a corresponding serum titer against SEL1L3 and the other expressing a BCR of unknown antigen specificity. The SEL1L3-reactive PVRL patient unfortunately relapsed with extra-nodal manifestation in the breast. Interestingly, the BCRs cloned and expressed both from the eye and the specimen obtained at relapse showed binding to SEL1L3, which is indicative of an ongoing dependence on antigen stimulation of the tumor. Moreover, with the detection of the same VH and VL genes in both manifestations the clonal origin could be confirmed. When comparing the BCR sequences of both manifestations with each other, it is noticeable that they largely match in the CDR3 region with numerous divergences outside of CDR3 suggesting dependence of the lymphoma cells on antigen, i.e. SEL1L3, stimulation. De-glycosylation experiments were performed on LCL cells derived from this patient. Interestingly, LCL cells derived from the SEL1L3-reactive PVRL patient showed binding to SEL1L3 antigen and could be killed with SEL1L3 incorporating immunotoxins. PCR analysis confirmed clonal B-cells among PVRL patient-derived LCL cells but no clonal relation to the malignant B cells found in the vitreous body or breast since the LCL clone used different VH and VL genes. Furthermore, NGS analysis of the LCL clone revealed no mutations commonly found in PVRL. The mechanisms for clonal selection of SEL1L3-reactive B-cells after EBV infection remain unclear but we believe that in this patient, due to hyper-N-glycosylation, an SEL1L3-directed immune reaction with a large polyclonal SEL1L3-reactive B-cell population was ongoing which resulted in PVRL development in the immune privileged site of the eye and later in selection of a different clone after EBV infection ex-vivo. These findings provide further evidence that lymphomas of the CNS, eyes, and testes are systemic in origin and transform only secondarily in immune-privileged sites. It has to be conceded that the frequency with which SEL1L3 could be identified as a BCR antigen of PVRLs is not particularly high (3/20), but we assume that further investigations will reveal even more autoantigens of PVRLs. SEL1L3 can therefore be seen as blueprint in its role as autoantigen in the development of PVRL.

Taken together, by investigating the antigen-reactivity of previously published BCR sequences of PCNSL und PVRL cases, SAMD14/neurabin-I could be confirmed as common antigen in PCNSL and SEL1L3 was identified as a common PVRL antigen supporting the hypothesis of chronic antigenic stimulation as pivotal part of PVRL genesis. In addition, functional assays with surrogate PVRL cell lines demonstrated the feasibility of potential therapeutic approaches incorporating SEL1L3.

### Supplementary Information

Supplementary Information.

## Data Availability

All data generated or analyzed during this study will be shared by the corresponding author upon reasonable request (moritz.bewarder@uks.eu).
