# Supplementary material for: Hyper-N-glycosylated SEL1L3 as auto-antigenic B-cell receptor target of primary vitreoretinal lymphomas
Source: Sci Rep. 2024 Apr 26;14:9571. doi: 10.1038/s41598-024-60169-5 (PMC11053041; doi:10.1038/s41598-024-60169-5)
Supplement: Supplementary file 1 — Supplementary Information. [file 41598_2024_60169_MOESM1_ESM.docx]

**Supplemental Figures**

*Original, uncropped Western Blot figures*

Suppl. Fig. 1: screening for antigenic PVRL BCR targets


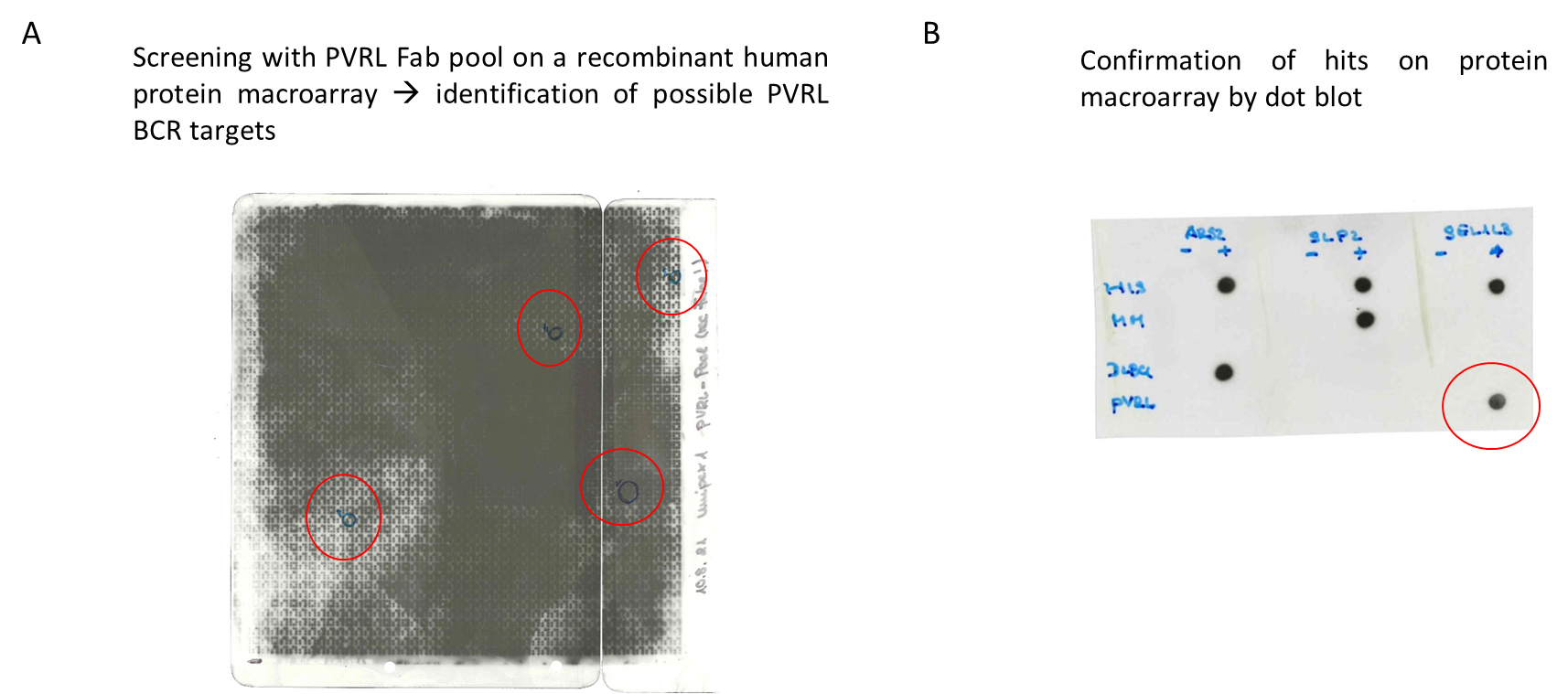


1. The PVRL Fab pool was used to identify PVRL BCR targets. To this end, pooled PVRL Fabs were incubated with a protein macroarray. The high density protein array consists of 37,200 independent clones derived from a human fetal brain cDNA expression library. For detection, an ECL chemiluminescence system was used. Positive signals (red circles) were localized according to the manufacturer's protocol. Corresponding clones were obtained from engine GmbH, Neuendorfstr. 17, 16761 Hennigsdorf, Germany. Recombinant proteins produced from these clones were used for further testing by dot blot, ELISA and functional assays.
2. Recombinant proteins are incubated with Fab pools. Positive control proteins ARS2 and SLP2 have previously been identified as targets of DLBCL BCRs and of myeloma paraproteins, respectively (left and middle column). His-tags are incorporated into recombinant proteins and reaction with anti-His antibodies serve as additional control (dots in upper row). SEL1L3 was confirmed as target of the PVRL Fab pool.

Suppl. Fig. 2 (Figure 2C, uncropped)

**OCI-Ly3 PVRL#22 (Fig. 2C, top left)**


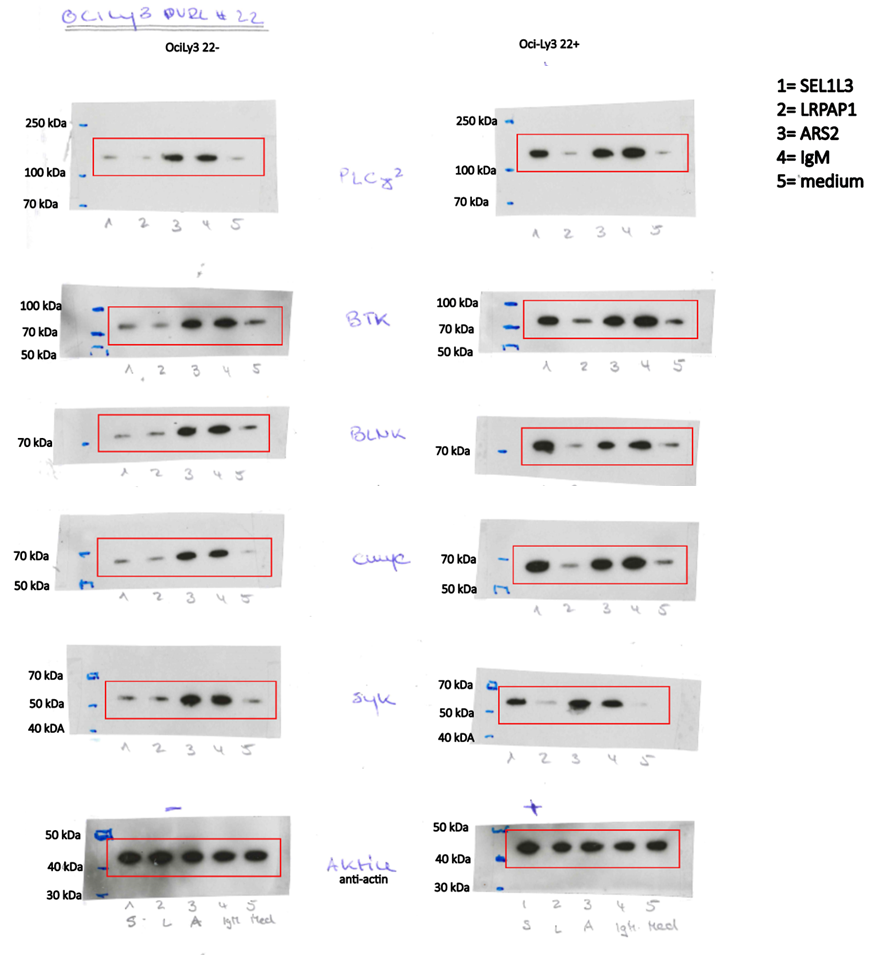


**TMD8 PVRL#22 (Fig. 2C, bottom left)**


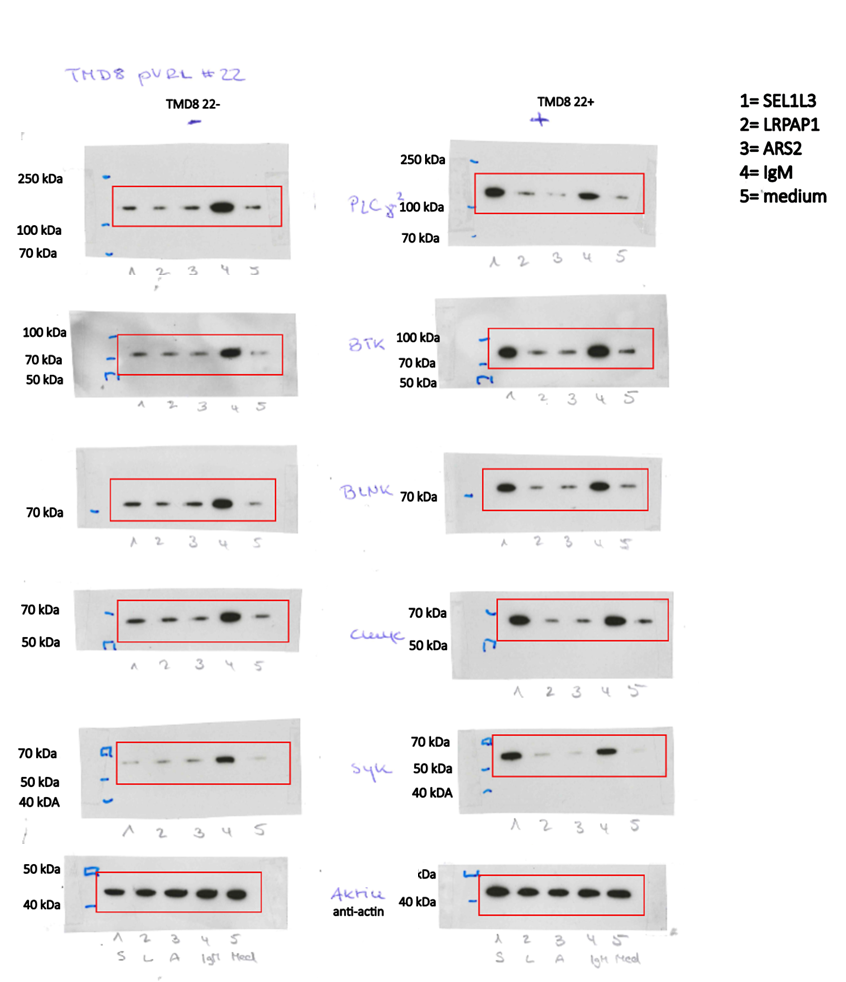


**OCI-Ly3 PVRL#30 (Fig. 2C, top right)**


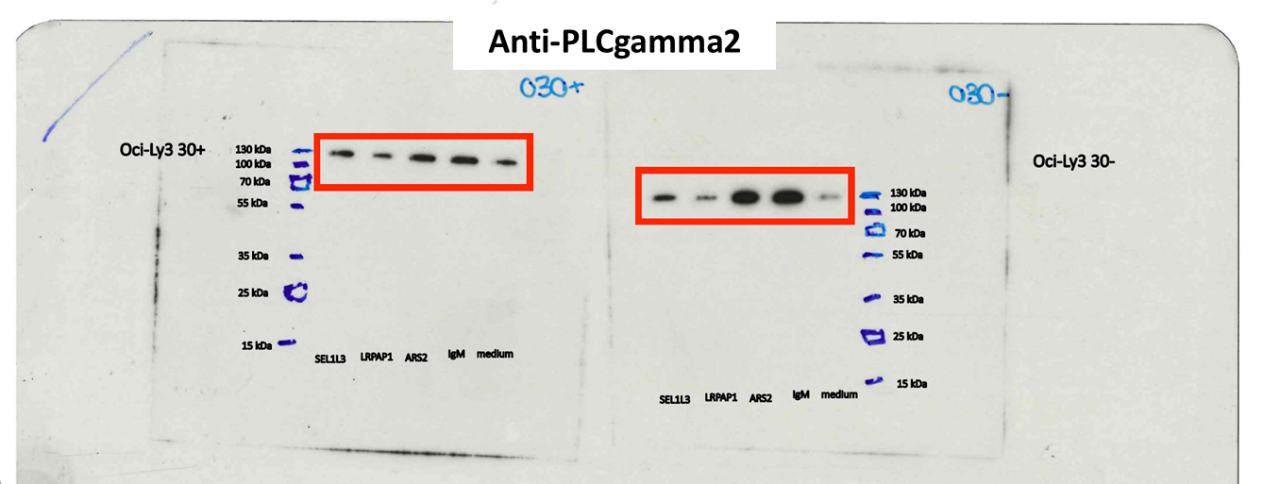


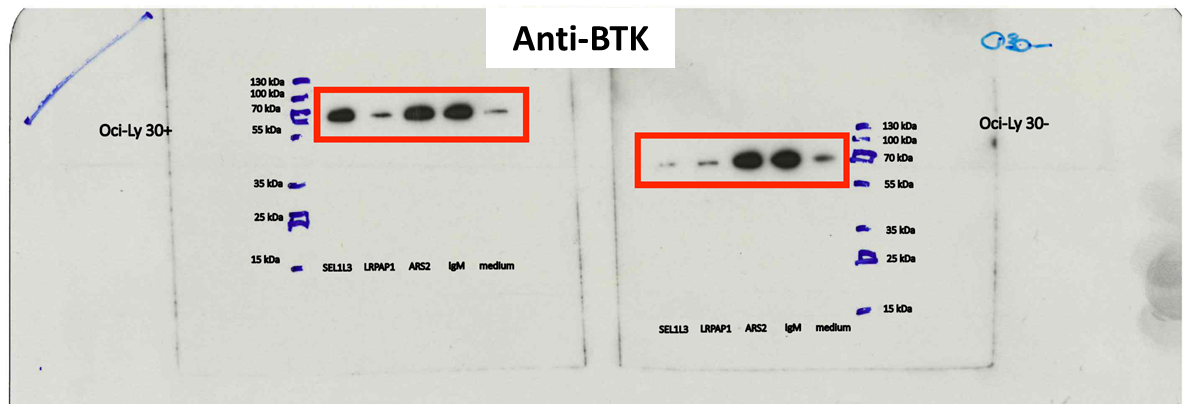


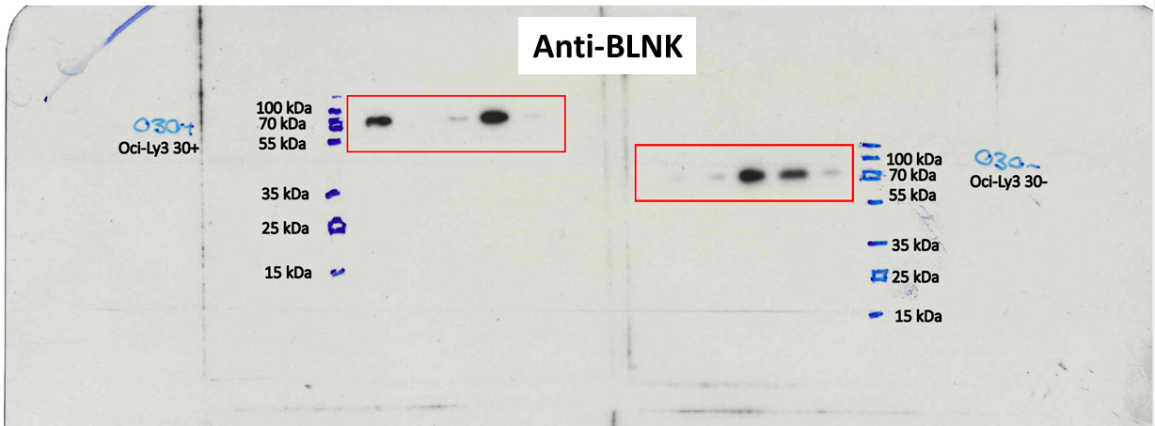


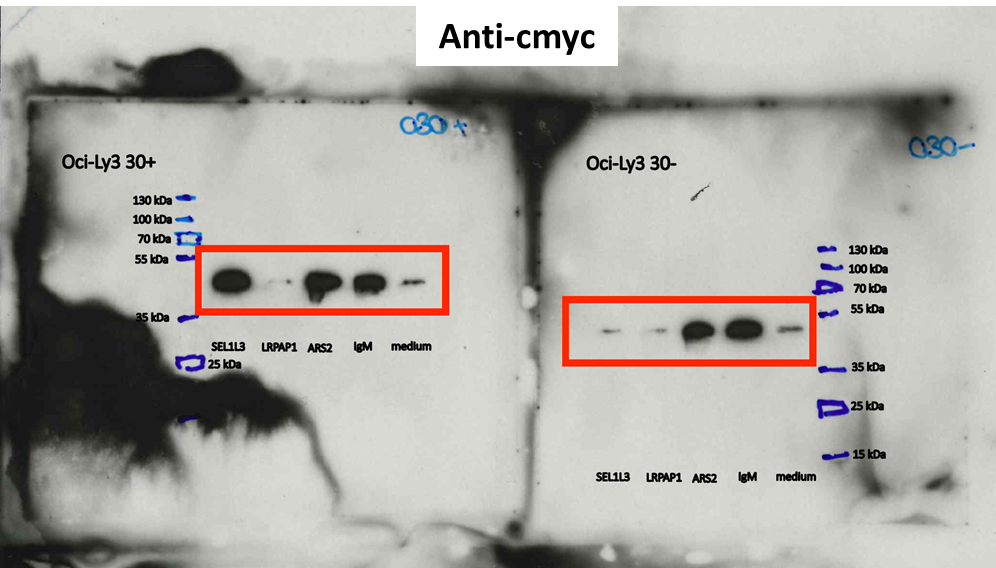


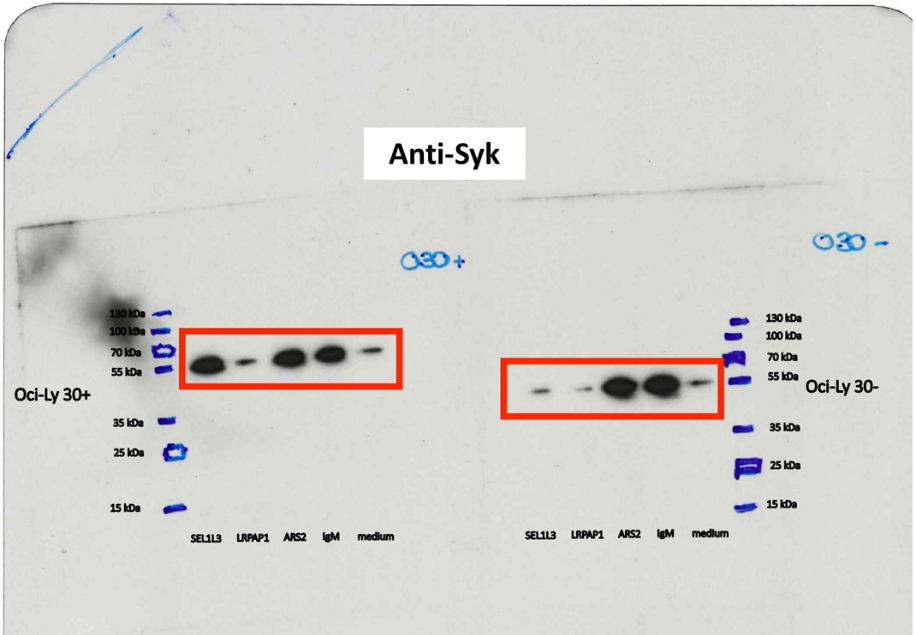


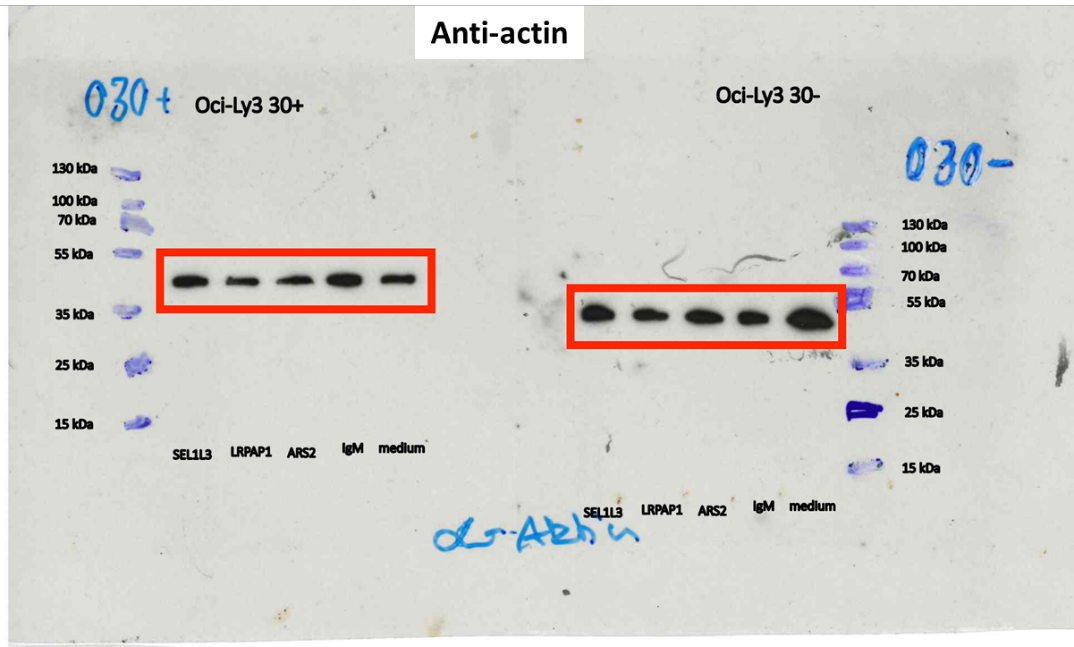


**TMD8 PVRL#30 (Fig. 2C, bottom right)**


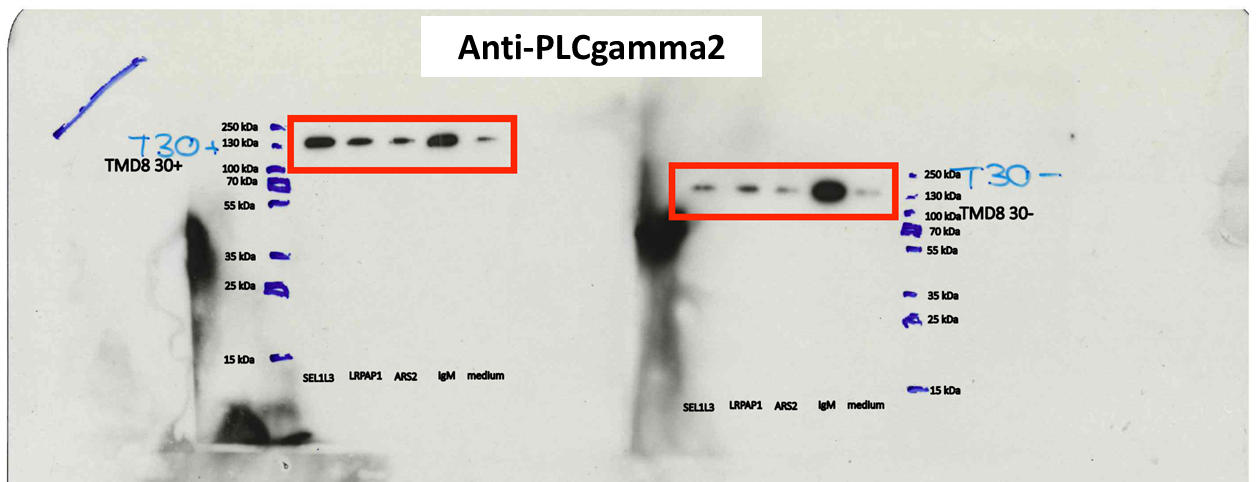


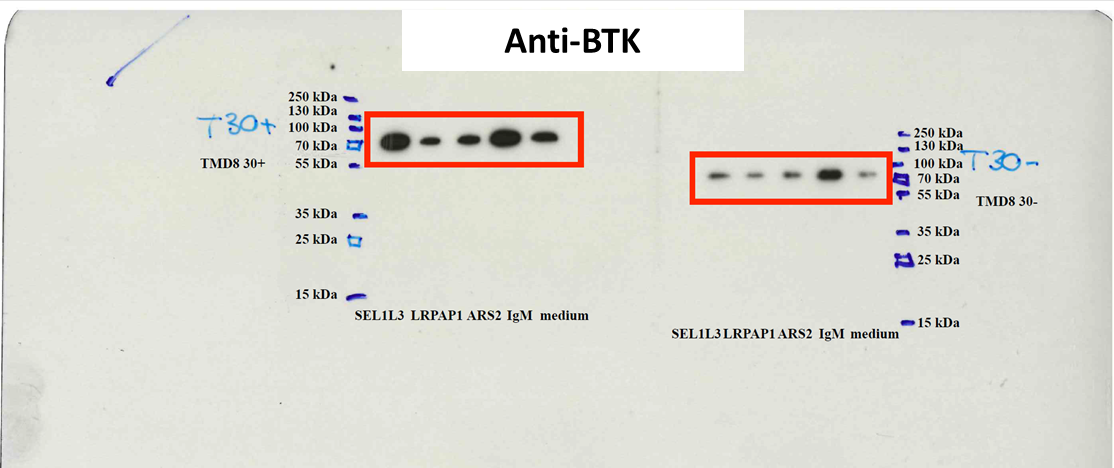


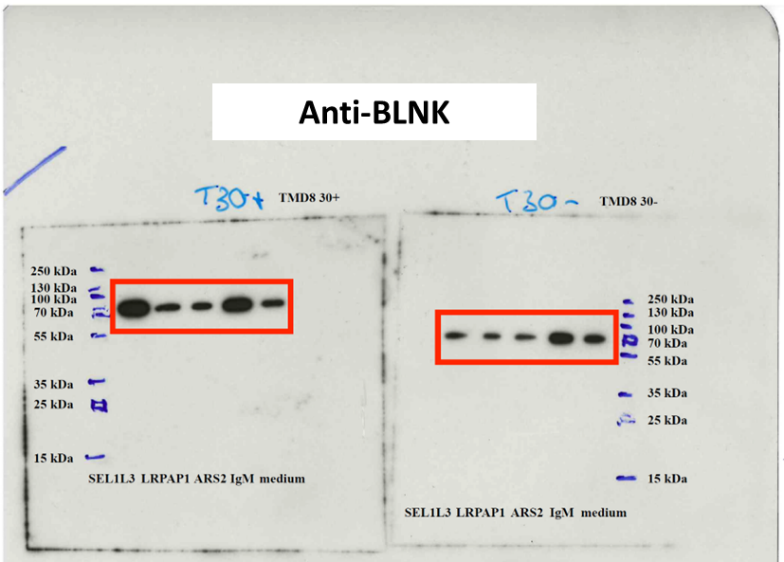


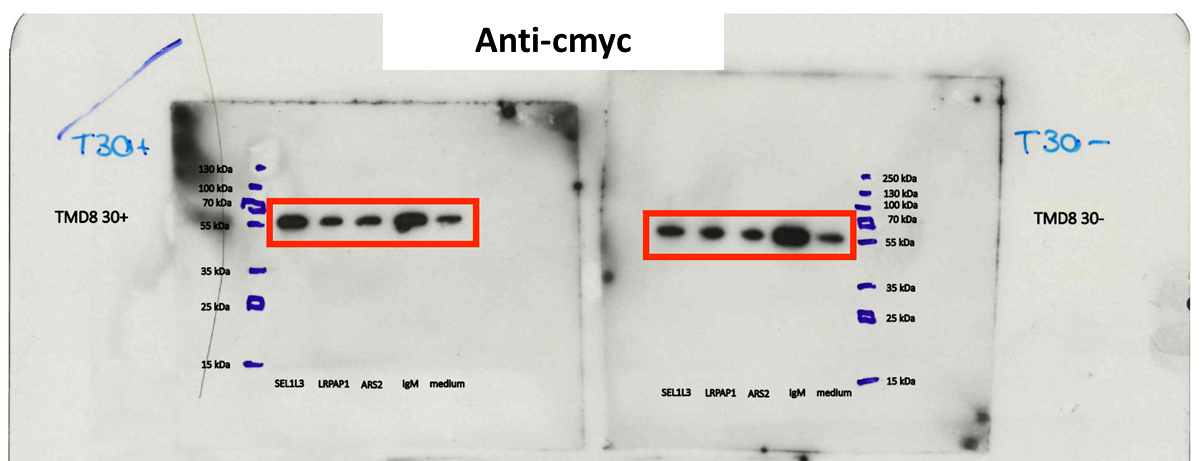


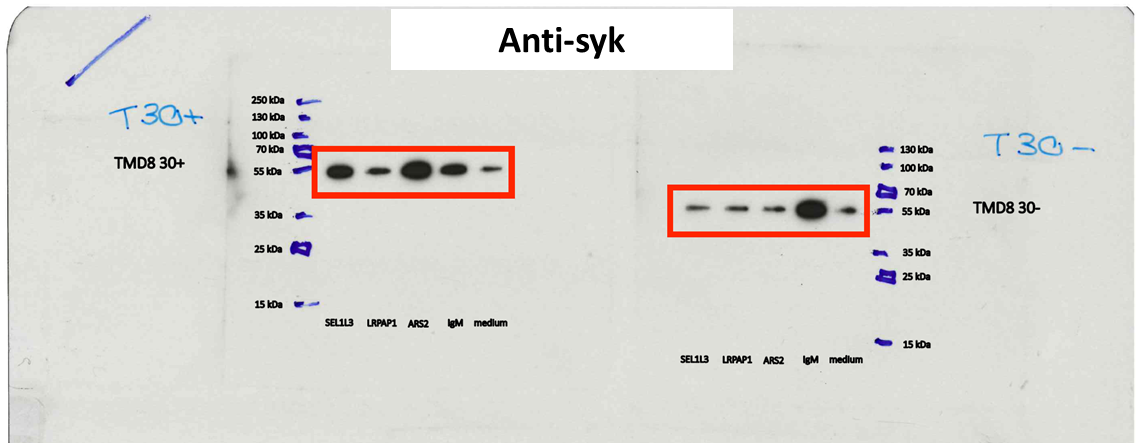


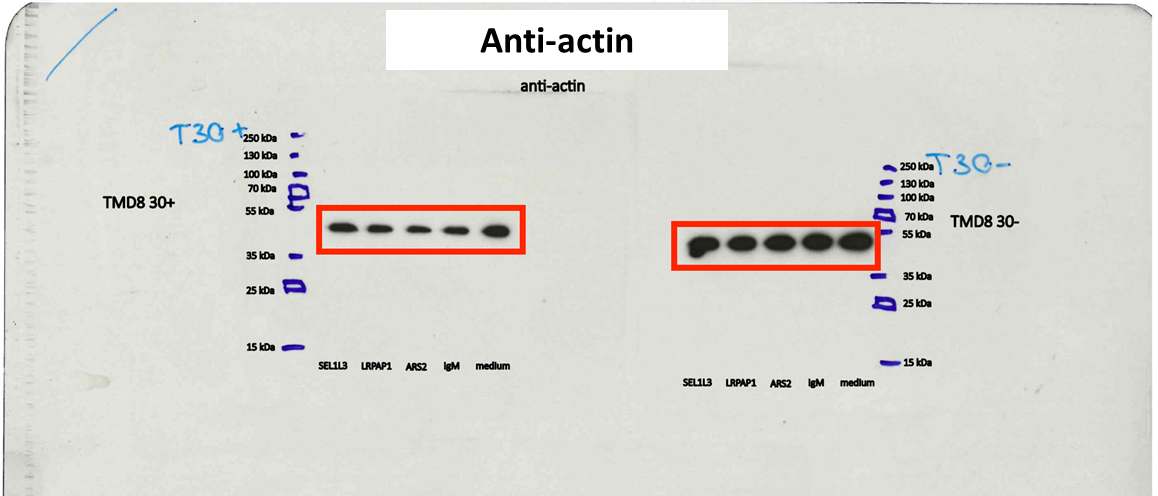


Suppl. Fig. 3 (Figure 4C, uncropped)


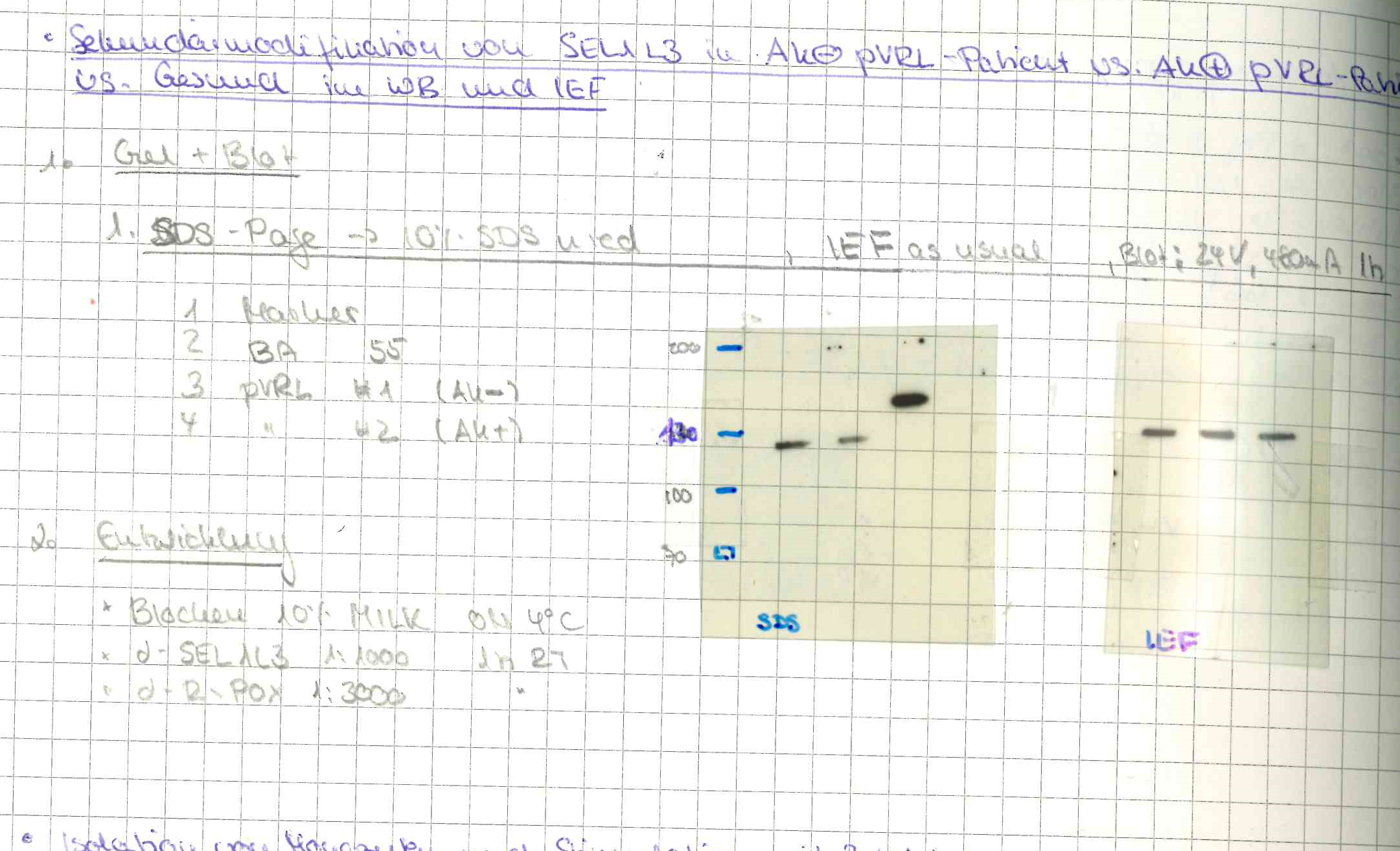


Suppl. Fig. 4 (Figure 4D, uncropped)


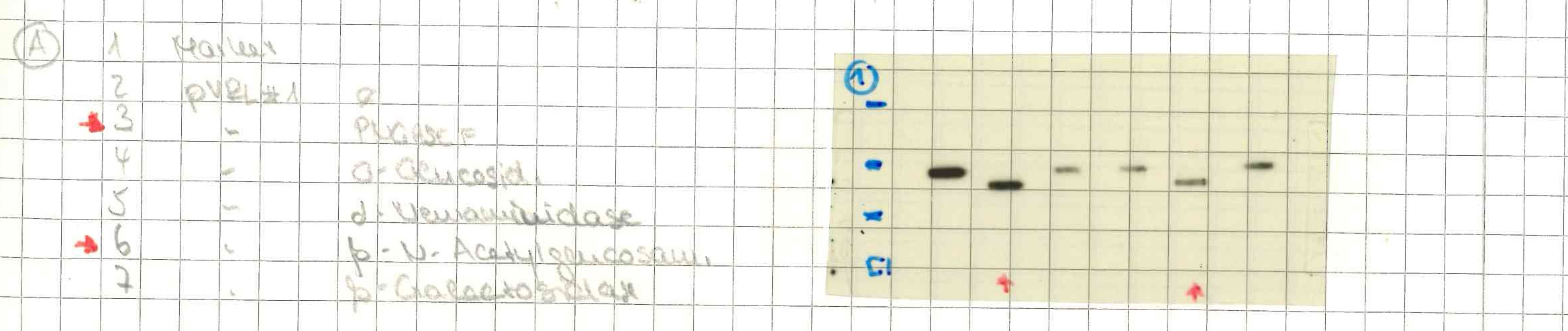


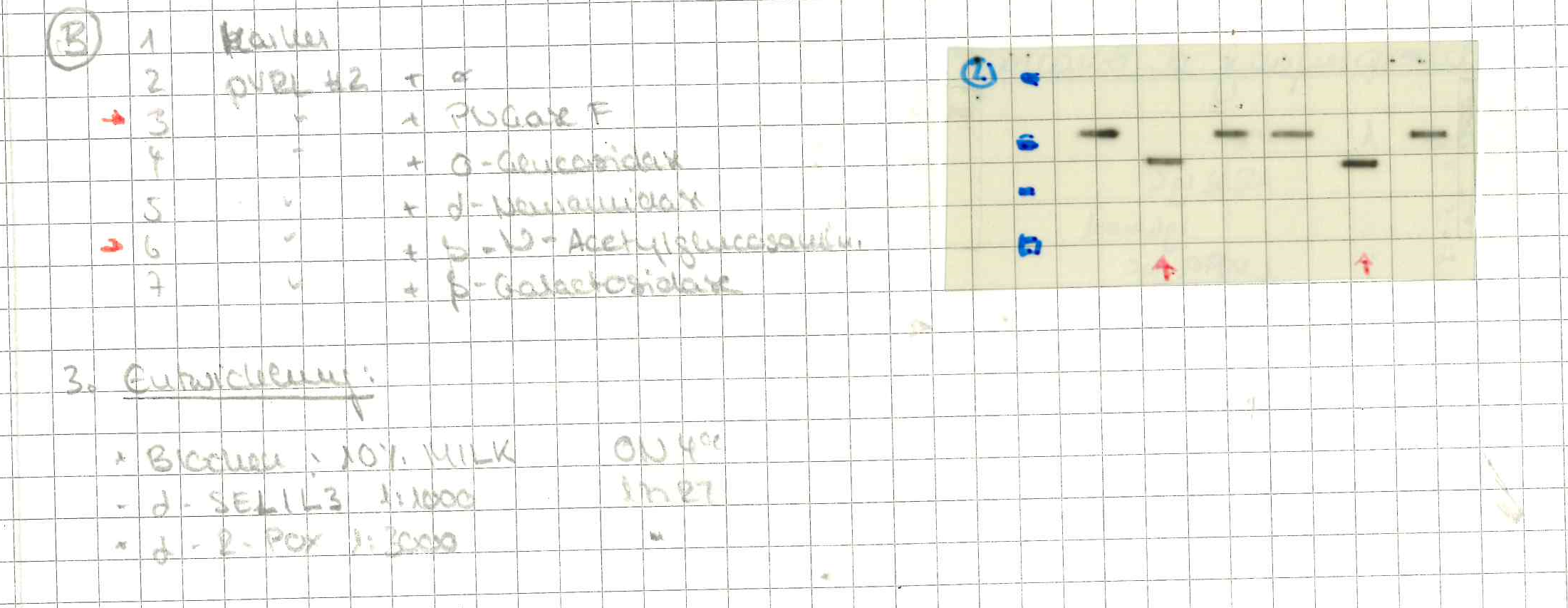


Suppl. Fig. 5 (Figure 4E, uncropped)


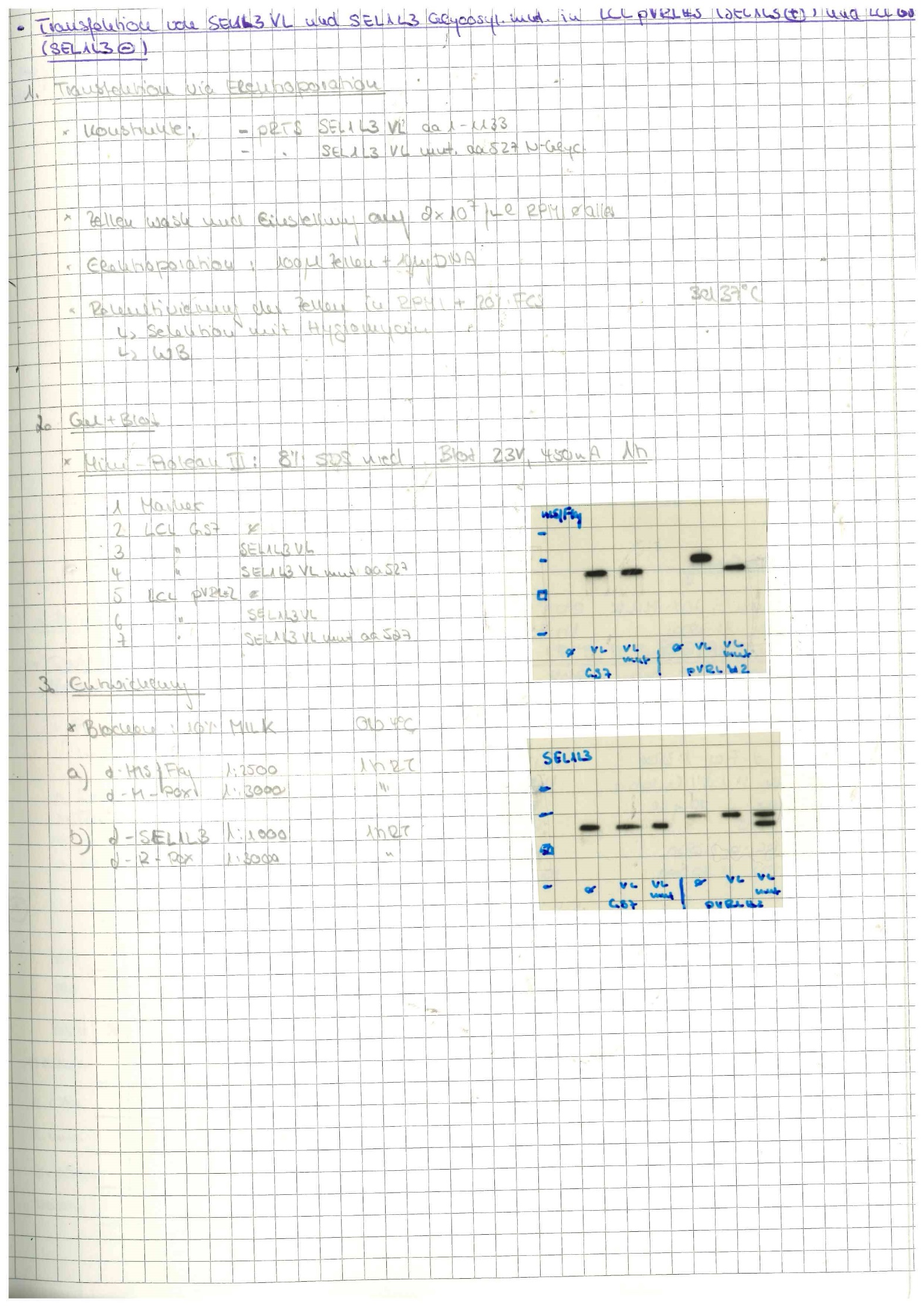


Suppl. Fig. 6 (Figure 5A, uncropped)


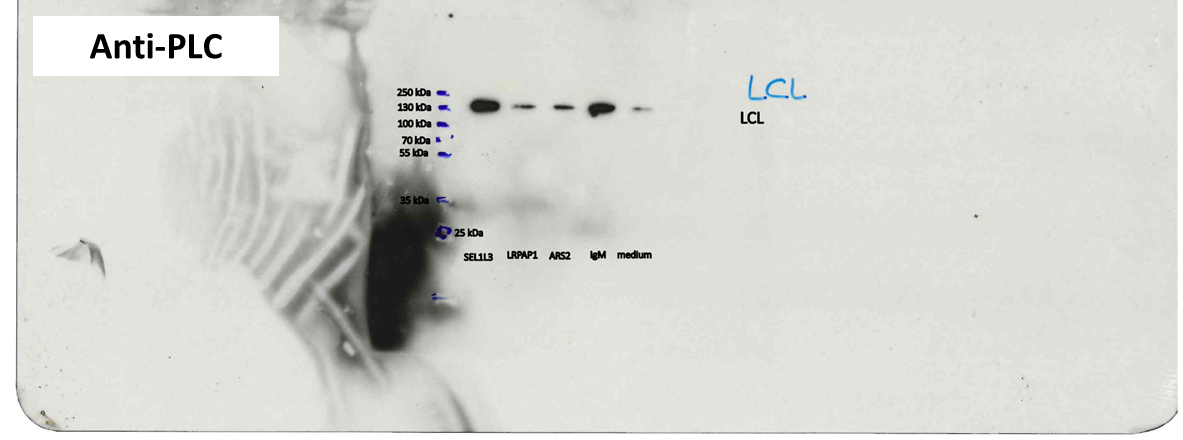


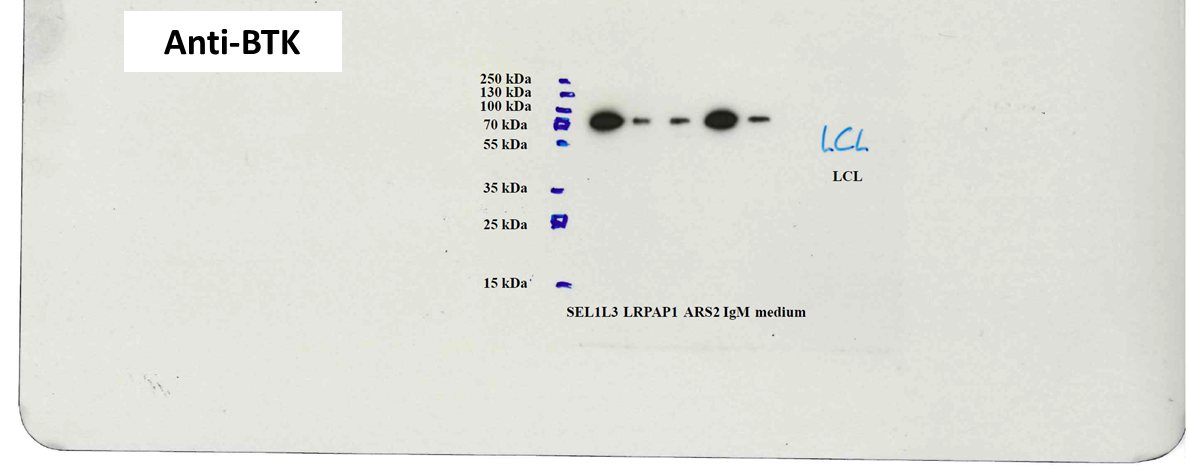


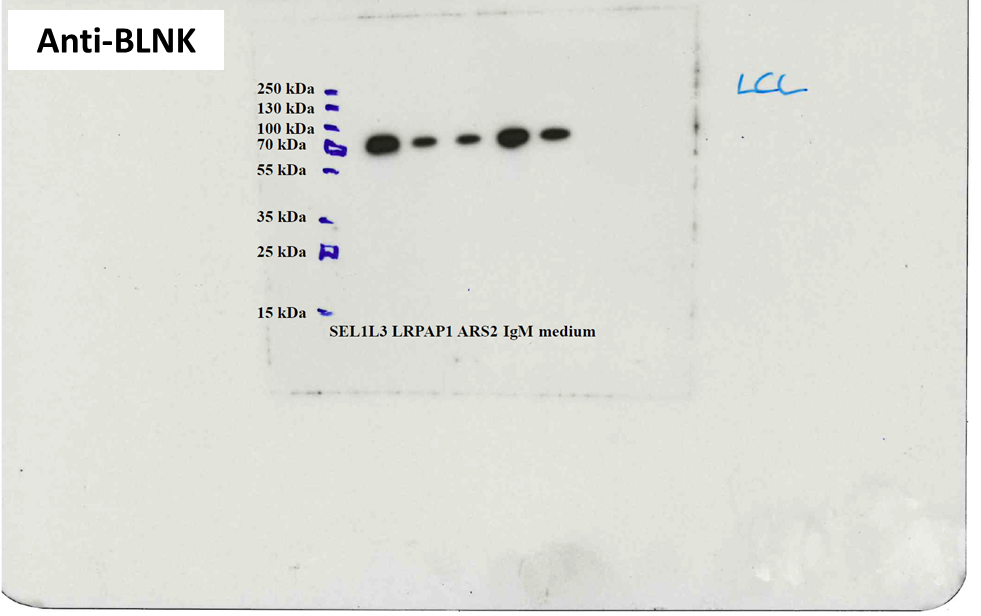


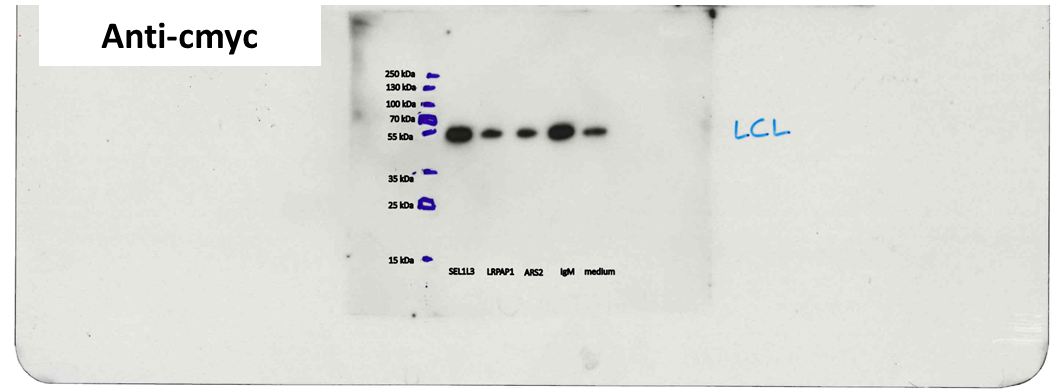


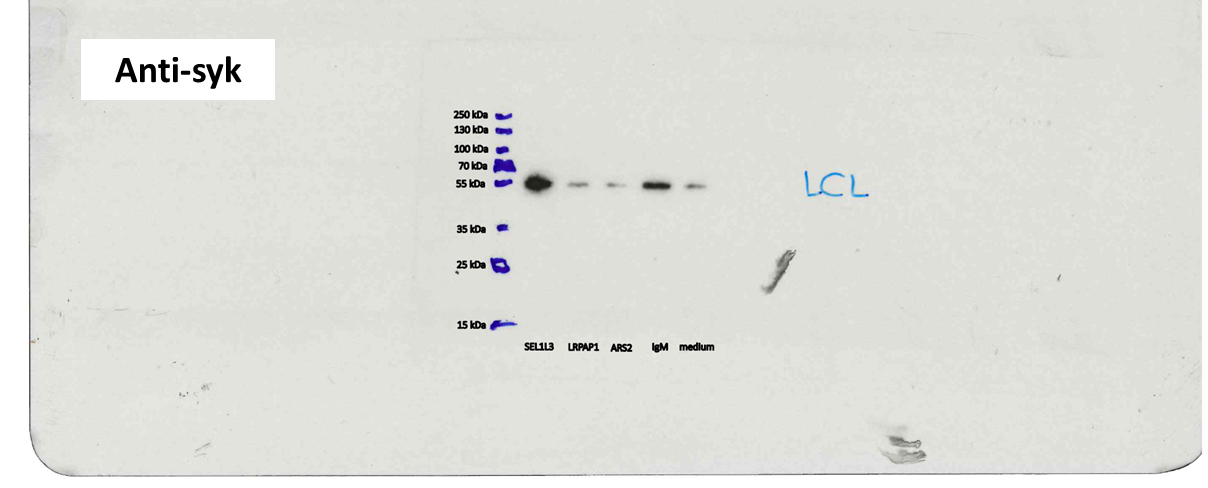


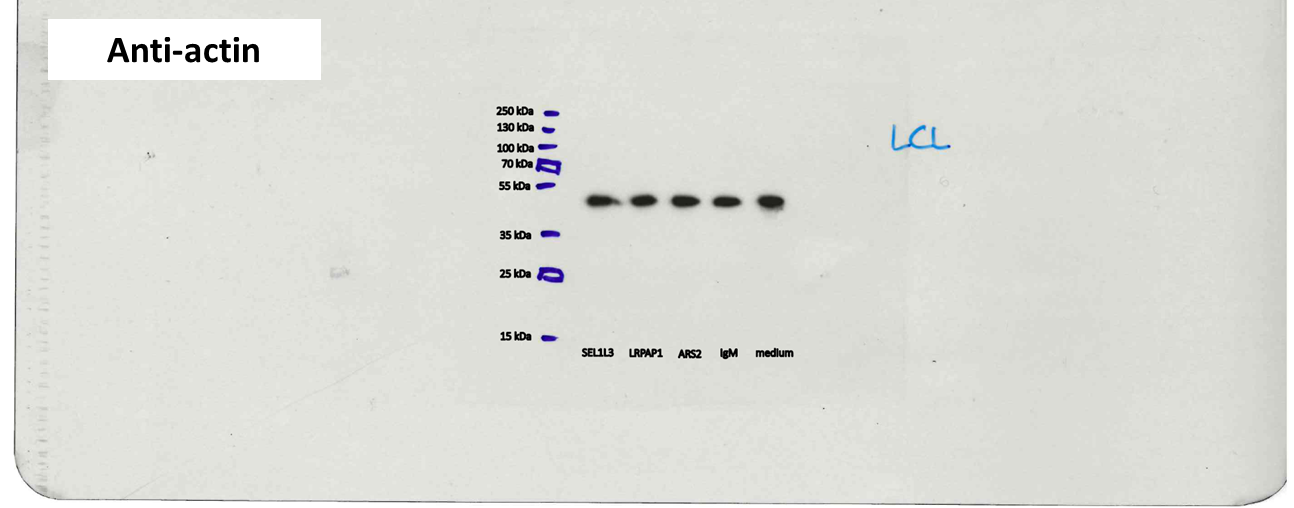


Suppl. Fig. 7 (Figure 5C, uncropped)

1)


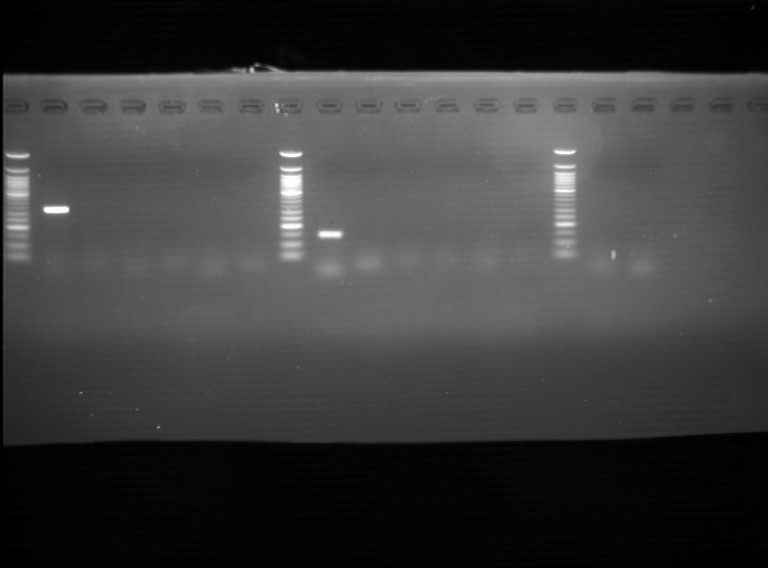


2)


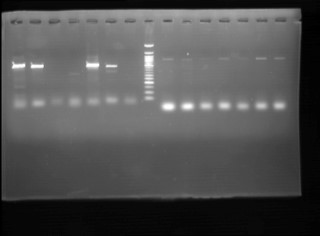


**Supplemental Tables**

**Suppl. Table 1: Quantitative flow cytometry data for figures 2A and 2B**

**Suppl. Table 2: Quantitative flow cytometry data for figures 3B**

**Suppl. Table 3: Quantitative flow cytometry data for figures 5B**

**Suppl. Table 4: Primers used for variable gene region PCRs**

**
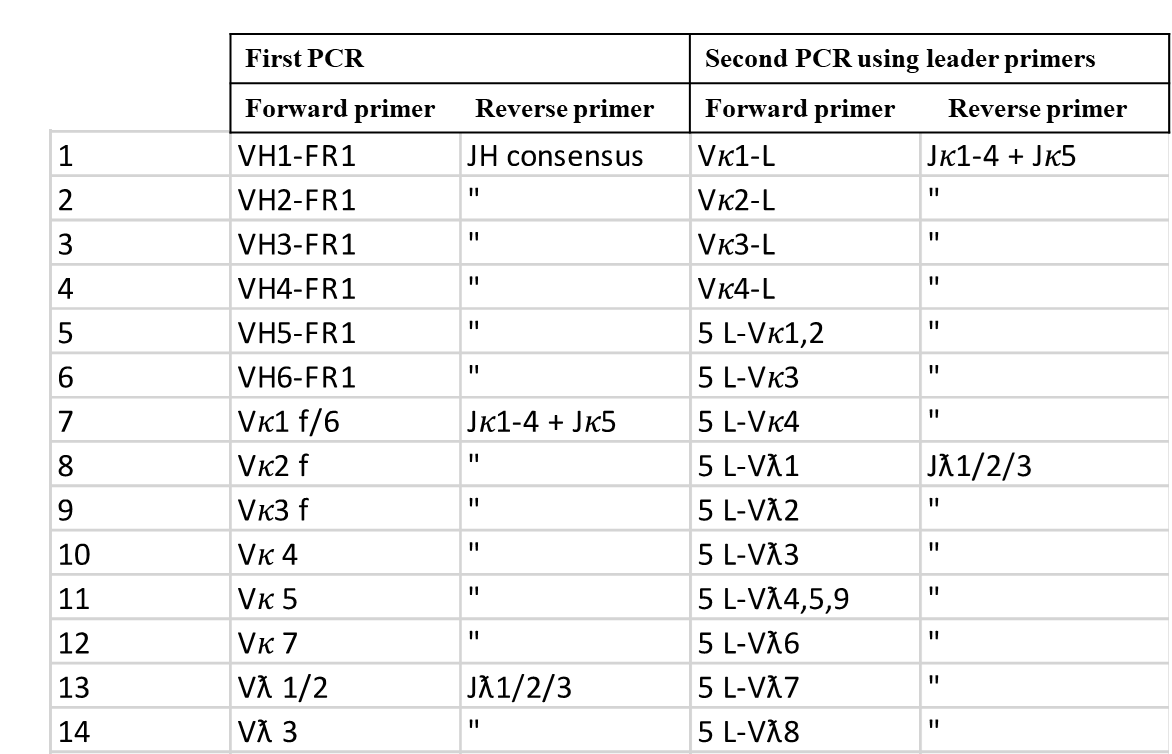
**

**Supplemental methods**

*ELISA for BCR reactivity against SEL1L3 and SAMD14/neurabin-I*

FLAG-tagged SEL1L3, SAMD14/neurabin-I and LRPAP1 epitopes, RpoC and galectin-3 were recombinantly expressed and added for 1h at room temperature (RT) in a concentration of 10 μg/ml to Nunc Maxisorb plates coated overnight at 4°C with murine anti-FLAG antibody (1:2,500, v\v, Sigma, Munich). Blocking was done with 1.5% (w/v) gelatin in TBS and washing steps were performed with TBS, 0.1% (v/v) and TritonX100. Recombinant PVRL and PCNSL Fabs (10 μg/ml) were added for 1h at RT. Subsequently, 3 washing steps with TBS/TritonX were performed and biotinylated goat anti-human IgG (heavy and light chain) (Dianova) was added at 1:2500 v/v for 1h at RT. For detection, after another washing step, peroxidase-linked streptavidin (Roche, 1:50,000) was used.

*Site-directed mutagenesis of wild type SEL1L3 and transfection of mutated SEL1L3 in LCLs*

Asparagine at position aa 527 is the closest N-glycosylation site to the identified PVRL BCR-binding epitope of SEL1L3. The QuickChange II Site-Directed Mutagenesis Kit (Stratagene) was used to mutagenize the amino acid asparagine to glutamine, disabling N-glycosylation. The SEL1L3 mutant was cloned into a pRTS vector containing a FLAG-tag and transfected into LCL cells derived from a healthy donor and from a PVRL patient with SEL1L3 serum autoantibodies (HD LCL and PVRL LCL #2 cells).

*Western blots of SEL1L3*

Lysates of lymphoblastoid cell lines (LCLs) of PVRL patients and healthy donors were loaded to a 12% and 8% sodium dodecyl sulfate-polyacrylamide gel, separated by electrophoresis, and transferred to a polyvinylidene difluoride membrane, using a transblot semidry transfer cell (Bio Rad). The membrane was blocked overnight at 4°C in PBS with 10% nonfat dry milk and transferred proteins were incubated with Rabbit IgG Anti-SEL1L3 antibody (ab154052, ABCAM, Cambridge, UK) at 1:500 for 1h or with murine anti-FLAG-antibody at 1:2000 for 1h, followed by 1h incubation at RT with goat HRP-labeled anti-rabbit antibody (Bio Rad) at 1:3000 for the anti-SEL1L3 antibody or by HRP-labeled anti-mouse-IgG antibody at 1:3000 for western blots with mutated FLAG-tagged fragments of SEL1L3. Chemiluminescence reagent was used for immunoblot visualization.

*Western blot of BCR pathway activation proteins*

For western blot analysis of proteins indicating BCR pathway activation, 5 x 10^4^ OCI-Ly3 and TMD8 cells transfected with inducible SEL1L3-reactive BCRs (BCR sequences were previously published by Belhouachi et al.) were treated with epitopes of the antigens SEL1L3, LRPAP1 and ARS2. Each antigen was used in a concentration of 2 µg/mL. Anti-IgM (2 µg/mL) served as positive control and medium as negative control. Doxycycline was used to induce SEL1L3-reactive BCR expression in OCI-Ly3 and TMD8 cells. It was added 1 day prior to antigen treatment at 1 µg/mL. Cells were incubated with antigens or anti-IgM for 5 days at 37 °C and 5% CO2. Subsequently, cell lysates were loaded to a 12 % sodium dodecyl sulfate-polyacrylamide gel, separated by electrophoresis and transferred to a polyvinylidene difluoride membrane.

Antibodies (rabbit) against pTyr525/526 SYK diluted 1:2000, pTyr759 PLCγ2 diluted 1:1000, pTyr223 BTK diluted 1:1000, and pTyr96 BLNK diluted 1:1000 (B-cell signaling sampler kit, CST), against actin diluted 1:2000 (Sigma), and murine antibody against MYC at a concentration of 1 µg/mL (Santa Cruz) were used as primary antibodies. Before incubation with primary antibodies, membranes were cut in order to allow for simultaneous western blot development. This was followed by washing steps and incubation with POX-conjugated anti-rabbit or anti-mouse antibodies diluted at 1:3000.

*Proliferation assays*

To assess proliferation of transfected DLBCL cell lines after the addition of antigens or immunotoxins, the EZ4U nonradioactive cell proliferation and cytotoxicity assay from Biomedica was used according to the manufacturer’s instructions. Specifically, 4 x 10^4^ cells per well were incubated with 2 µg/mL of SEL1L3 (aa406-604), SEL1L3 (aa 510-585), ARS2, LRPAP1, PC9, SamD14 or SLP2 for 3 days at 37°C and 5 % CO2. For the immunotoxins ARS2-ETA, RpoC-ETA and SEL1L3-ETA, a concentration of 1 µg/ml was used. All experiments were performed in triplicate, i.e. in three separate wells.

*Cytotoxicity assays*

Lactate dehydrogenase (LDH) release assays were used to demonstrate cytotoxicity of SEL1L3 immunotoxins. OCI-LY3 and TMD8 cells (5 × 10^3^/well) previously transfected with an inducible SEL1L3-reactive BCR were incubated with SEL1L3-ETA, ARS2-ETA and RpoC-ETA in a dilution series of 1 µg/ml, 0.5 µg/ml, 0.25 µg/ml and 0.125 µg/ml, or no immunotoxin in a 96-well plate. Percentage of specific lysis was determined as (experimental lysis − spontaneous lysis)/(maximum lysis − spontaneous lysis) × 100. 10% Triton X-100 was used to determine maximum lysis. LDH release was measured according to protocol of the LDH release assay kit (Cytotoxicity Detection KitPLUS™, Roche, Mannheim, Germany). After adding stopping solution, plate read-out was performed in a microplate reader at 490 nm (Victor II, PerkinElmer, Rodgau, Germany). All experiments were performed in triplicate, i.e. in three separate wells.

*Flow cytometric apoptosis and necrosis assays*

For the analysis of apoptosis and/or necrosis the Annexin V-FITC Apoptosis Detection Kit (Sigma-Aldrich) was used. In short, 1 × 10^6^ cells/mL OCI-LY3, TMD8 or LCL cells stably transfected to express a BCR with reactivity against SEL1L3 were treated with ARS2-ETA, RpoC-ETA, SEL1L3-ETA (all at 0.5 µg/mL), staurosporine (1 µg/mL), or medium only for 24 hours at 37°C, 5% CO2. The next day, cells are washed twice in 1 ml PBS and resuspended in 500 µl binding buffer. 5 µl Annexin-V-FITC and 10 µl of propidium iodide were added and incubated for 10 min at room temperature, followed by flow cytometric analysis.
